# Deep mantle plumes feeding periodic alignments of asthenospheric fingers beneath the central and southern Atlantic Ocean

**DOI:** 10.1073/pnas.2407543121

**Published:** 2024-11-05

**Authors:** Federico D. Munch, Barbara Romanowicz, Sujoy Mukhopadhyay, Maxwell L. Rudolph

**Affiliations:** ^a^Institute of Geophysics, ETH Zurich, Zurich 8092, Switzerland; ^b^Department of Earth and Planetary Science, University of California, Berkeley, CA 94720; ^c^Institut de Physique du Globe, Paris 75238 CEDEX 05, France; ^d^Department of Earth and Planetary Sciences, University of California, Davis, CA 95616

**Keywords:** full waveform seismic imaging, mantle dynamics, midplate volcanism, radiogenic isotopes, seismic tomography of the earth’s mantle

## Abstract

High-resolution full waveform seismic tomography of the Earth’s mantle beneath the south and central Atlantic Ocean brings into focus a series of asthenospheric low shear velocity channels, or “fingers” on both sides of the southern and central mid-Atlantic ridge (MAR), elongated in the direction of absolute plate motion with a spacing of ∼1,800 to 2,000 km, and associated with bands of shallower residual seafloor depth anomalies that suggest channeled flow over thousands of kilometers. Each of the three most clearly resolved fingers on the African side of the MAR corresponds to a separate group of whole mantle plumes rooted in distinct patches at the core–mantle boundary, feeding hotspots, and volcanic lines with distinct isotopic signatures. Plumes of a given group appear to merge at the top of the lower mantle before separating again, suggesting interaction of deep mantle flow with a more vigorous mesoscale circulation in the upper mantle. The corresponding hotspots are generally offset from the location of the deep mantle plume roots. The distinct isotopic signatures of these hotspot groups are also detected in the mid-ocean ridge basalts at the location where the fingers meet the ridge. Meanwhile, at least some of the variability within each plume group could originate in the upper mantle and extended transition zone where plumes in a given group appear to merge and pond. This study also adds to mounting evidence that the African large low shear velocity province is not a uniform, unbroken pile of dense material rising high above the core–mantle boundary, but rather a collection of mantle plumes rooted in patches of distinct composition.

## Significance

Several lines of mid-plate volcanoes on each side of the mid-Atlantic ridge in the south-central Atlantic are thought to be related to plumes of hot material rooted deep in the Earth’s mantle, but cannot be explained by the classical plume model because of the lack of a clear age progression in several of them. Full waveform seismic tomography shows that these volcanic lines are the manifestation of active flow in periodically spaced channels beneath the lithosphere, each associated with a separate group of mantle plumes rooted at the core–mantle boundary, with different geochemical signatures. This study clarifies some of the pathways of upwellings from the deep mantle, including the presence of mesoscale dynamics in the upper ∼1,000 km.

Many questions remain about the physical mechanisms that couple the large-scale global mantle circulation and the motions of tectonic plates (1), and in particular, about the role of deep mantle plumes in global dynamics, and their interaction with mesoscale circulation in the upper third of the mantle (2). In the last decade, the advent of global scale whole mantle full waveform inversion (FWI) has made it possible to shed some light on the Earth’s plumbing system. In the lower mantle, broad, vertically oriented low-velocity conduits, rooted at the core–mantle boundary (CMB) have been imaged beneath major hotspots and interpreted as deep mantle plumes (3, 4). In the upper mantle, global FWI (5) revealed a system of horizontally elongated low-velocity channels quasi-periodically aligned with the absolute plate motion (APM) with a spacing of ∼1,800 to 2,000 km beneath the Pacific Ocean basin, and suggested such channels may also be present in the Indian and Atlantic basins, although the latter were not as clearly resolved. Targeted seismic tomographic studies based on accurate numerical wavefield computations and FWI across the entire depth of the mantle beneath ocean basins can further help address these questions by providing images of present-day mantle structure at increasingly high resolution.

Building upon our previous FWI modeling of shear velocity and radial anisotropy at the global scale (6), and considering a larger dataset of regional three-component seismic waveforms, we have obtained a higher-resolution whole mantle model of the central and southern Atlantic ocean (SEMATL_23), including significant portions of the bordering African and south-American continents. This model provides further insights into the possible organization of flow across the mantle. Details on the construction of the model can be found in *Methods* and in *SI Appendix*, Figs. S1–S4. In what follows, we describe the salient features of the isotropic part of model SEMATL_23: low-velocity channels in the asthenosphere corresponding to separate deep mantle plume groups. We relate them to trace isotopic signatures of the corresponding hotspot volcanoes and discuss their geodynamic implications.

## Upper Mantle Structure

As is well known, the isotropic shear-wave velocity ($V_{s}$) in the uppermost mantle reflects both compositional and thermal heterogeneity that can be directly related to known tectonic processes. Down to ∼100 km depth, it is dominated by a narrow low shear velocity band beneath the mid-Atlantic ridge (MAR) and fast continental roots on the African and South American side, consistent with many other tomographic models (*SI Appendix*, Fig. S5). The MAR signature at 50 km is stronger than in the starting model SEMUCB_WM1(6), extends down to depths of ∼150 km and disappears at larger depths. In contrast to the MAR signature, the fast cratonic roots in western and southern Africa and in northern South America extend down to 200 to 250 km depth (*SI Appendix*, Fig. S5). Notably, two zones of faster than average $V_{s}$ are present down to at least 150 km offshore, one west of Africa (labeled A in *SI Appendix*, Fig. S5*A*) and the other east of South America (labeled B in *SI Appendix*, Fig. S5*A*). The latter features are present in some other global and regional models and have been interpreted as representing remnants of delaminated continental lithosphere left behind during ocean spreading (e.g., ref. 12).

**Fig. 1.**
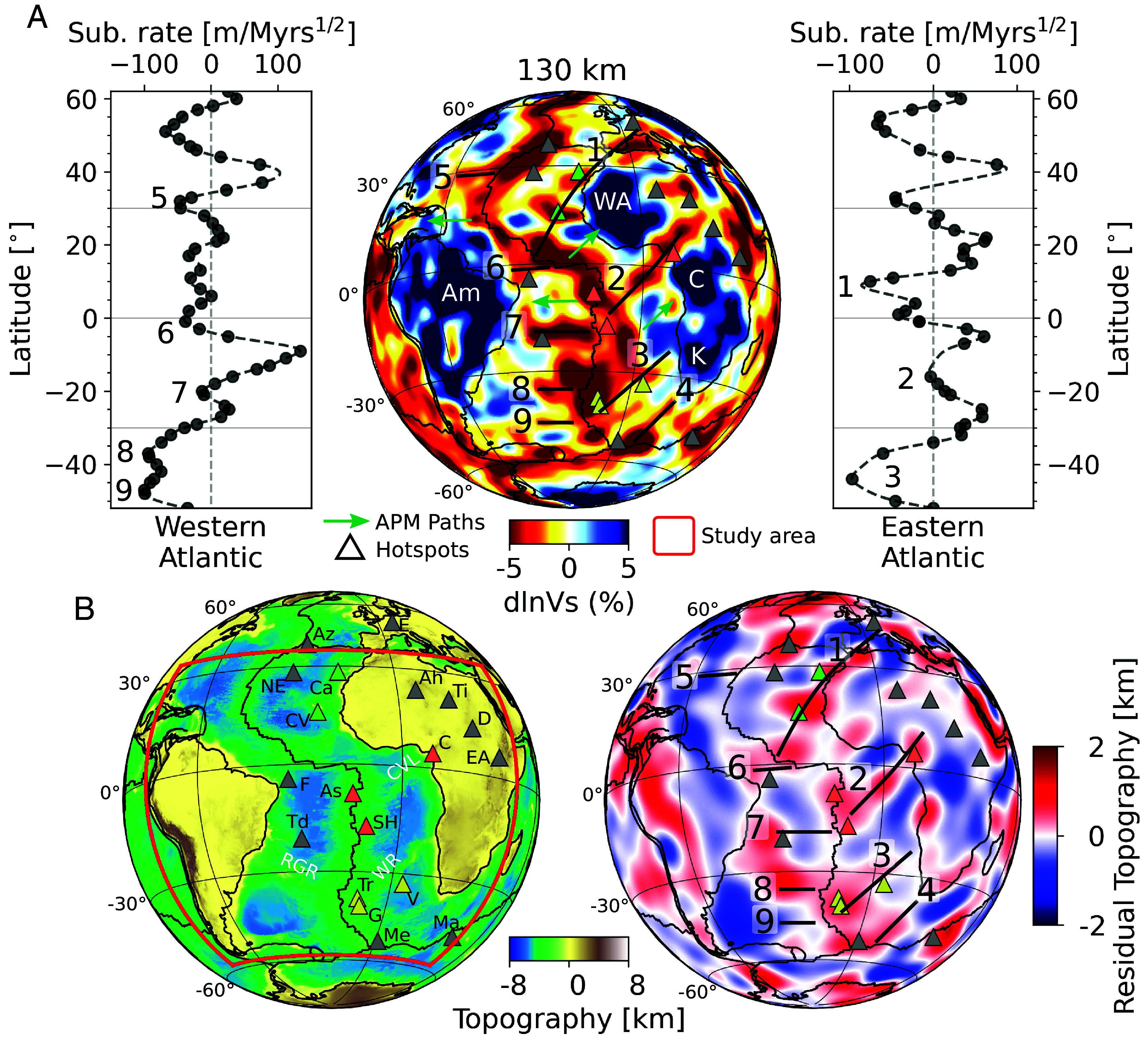
Upper mantle mesoscale seismic structure, bathymetry, and dynamic topography. (*A*) *Center*: Map of relative variations in shear-wave velocity (dlnVs=$\frac{V_{s}-V_{s}^{\mathrm{ref}}}{V_{s}}$, in percent) at a depth of 130 km in model *SEMATL_23*. Outside the target region (red box in panel *B*), the model is *SEMUCB_WM1* (6). Triangles show the location of major hotspots (7) color-coded based on their similarities in isotopic signature (Fig. 2). Green arrows and black segments indicate the direction of absolute plate motion (APM; 8), outside and within the low-velocity channels (LVCs) marked by numbers. *Left/Right:* subsidence rates as a function of latitude on the western/eastern side of the MAR, calculated along flow lines in the spreading direction [from Calcagno and Cazenave (9)]. Note the long wavelength component (>8,000 km) has been removed. Numbers (1 to 9) associate each of the LVCs with minima in the subsidence rate on each side of the MAR. Not numbered minima in the subsidence rates at latitudes higher than $30^{{}^{\circ}}$N may also correspond to fingers but are outside the scope of this study. (*B*) *Left*: map showing topography and location of hotspots discussed in the text. As, Ascension; Ah, Ahaggar; Az, Azores; Ca, Canary; C, Cameroon; CV, Cape Verde; D, Darfur; E, Eiffel; EA, East Africa F, San Fernando; G, Gough; Ma, Marion; Me, Meteor; NE, New England; SH, Saint Helena; Td, Trindade; Ti, Tibesti; Tr, Tristan da Cunha; V, Vema. Major volcanic seamount chains are noted in white: RGR, Rio Grande Rise; WR, Walvis Ridge; CVL, Cameroon volcanic line. The red line indicates boundary of the region covered by the new model *SEMATL_23*. *Right*: Residual topography (i.e., proxy of dynamic topography) from Hoggard et al. (10) filtered between harmonic degrees 4 and 30, with the same labeling of APM and channel numbering as in panel (*A*). Correlation coefficients between shear-wave velocity variations and dynamic topography by Hoggard et al. and a more recent study by Holdt et al. (11) are shown in *SI Appendix*, Fig. S6.

Interestingly, at asthenospheric depths beneath the oceanic lithosphere (depths > 50km), the most prominent feature in model SEMATL_23 is a series of horizontally elongated low-velocity channels (LVCs), originating near the MAR with a relatively regular spacing of ∼2,000 km (Fig. 1*A*), similar to that observed in the young parts of the Pacific Ocean (5), and extending on both sides of the MAR in a direction close to the corresponding APM. These channels are most prominent in the depth range 80 to 150 km, which is somewhat shallower than in the Pacific where they culminate in the depth range 200 to 250km.

On the eastern side of the MAR, these LVCs extend in a SW to NE direction close to the APM direction (8) of the African plate: from Cape Verde toward Canary and the Mediterranean (#1), from St Helena/Ascension toward and beyond Cameroon (#2), along the Tristan/Gough/Vema line (#3) and from Bouvet toward the tip of Africa (#4) (Fig. 1*A*). We note that the latter two LVCs are somewhat more clearly expressed in model *SA2019* (13). On the western side of the MAR, the LVCs extend in an E-W direction, slightly tilted toward the NE, close to the APM of the south-American plate: along the New England seamount line (#5), along the central east-west segment of the MAR toward Fernando (#6, slightly north of the hotspot), from the MAR toward Trindade (#7), and in the Rio Grande Rise region (#8). LVC #9, in the southernmost western Atlantic, is not as clear as others, possibly due to the lower resolution of the model at its southernmost edge. All of these features (except perhaps for LVC #9) lie beneath mid-plate volcanic lines and seamounts, many of which do not have clear age progressions as would be expected from the classical hotspot track model (14). The remarkable correlation of low shear velocities with minima in subsidence rate measured at the MAR along flow corridors (Fig. 1*A*) and regions of dynamic uplift in a recent study of residual topography (Fig. 1*B*) points to a dynamic origin in the convective upper mantle, that is at least partially distinct from processes occurring at the MAR, for which the correlation between seismic structure and residual topography is stronger, but peaks at shallow depths, and more rapidly decreases below ∼100 km depth (*SI Appendix*, Fig. S6).

## Relation to Deep Mantle Plumes

We acknowledge that some of the LVCs are not as clear as others, especially on the western side of the MAR, where, for example, LVC #6 may be difficult to distinguish from the MAR-related signal, even though it extends deeper, and LVC #7 does not have an expression in the recent residual topography map, although it is associated with a minimum in the subsidence rate of Calcagno and Cazenave (9). In what follows, we focus on the eastern side of the MAR, which has been more extensively studied by geologists and where the orientation of the LVCs is more clearly distinct from that of fracture zones, oriented more in the East-West direction. Whether the volcanic and seamount chains observed in the eastern Atlantic and western Africa are fed by individual mantle plumes and what their relation might be to the African large low shear-velocity province (LLSVP), are two questions widely debated in the literature. Here, we show that each of the three main uppermost mantle LVCs on the eastern side of the MAR is associated with its own deep mantle plume or plume group and that these plume groups are well separated from each other all the way down to the CMB.

**Fig. 2.**
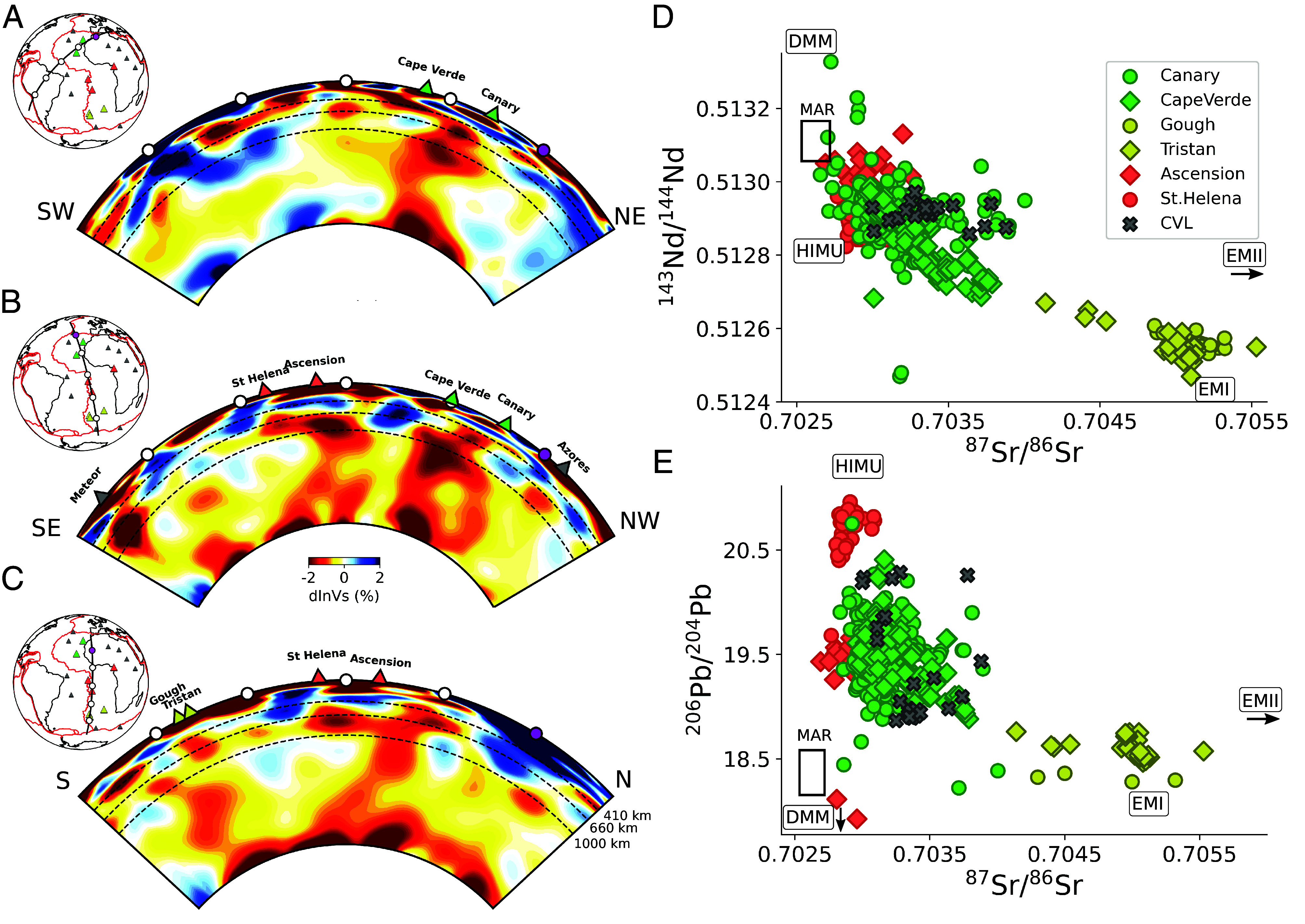
(*Left*) Whole-mantle depth cross-sections showing shear velocity perturbations with respect to the 1D regional average (dlnVs, in percent) in model SEMATL_23, in the vicinity of major hotspots (triangles). Note that the structure is 3D across the whole mantle, so that the plume conduits in the lower mantle do not generally plot exactly beneath the corresponding hotspots. (*A*) Cross-section oriented so as to showcase the Cape Verde-Canary plume group #1. (*B*) NW-SE cross-section showing the separation across the whole mantle of the Ascension-St Helena plume group #2 and the Cape Verde-Canary plume group #1. (*C*) North–South cross-section showing a plume conduit beneath Tristan (#3), well separated from plume group #2 across the lower mantle. (*Right*) Radiogenic isotope anomalies in (*D*) ^87^Sr/^86^Sr and ^143^Nd/^144^Nd space and (*E*) ^87^Sr/^86^Sr and ^206^Pb/^204^Pb space for major hotspot groups discussed in the text. This system was chosen because abundant data are available for all hotspots considered here. The data were downloaded from the Georoc database (https://georoc.mpch-mainz.gwdg.de/georoc/) (15) on March 9, 2024. Data for the CVL was extracted from the compilation of Stracke et al. (16). Colors (red, green, and yellow) indicate hotspot groups. Different shapes within each group correspond to individual hotspots. Rectangles represent Atlantic MORB (MAR) and reference values for DMM, HIMU, EMI, and EMII (17), with black arrows indicating that the end-member lies outside the range depicted in the panel. Note that the Cameroon volcanic line (CVL) signature (crosses) is distinct from that of the Tristan/Gough group, and closer to that of Ascension/St Helena with possible contamination from the African subcontinental lithosphere, in agreement with model SEMATL_23 showing a possible connection to plume group #2 (e.g., Fig. 5).

**Fig. 3.**
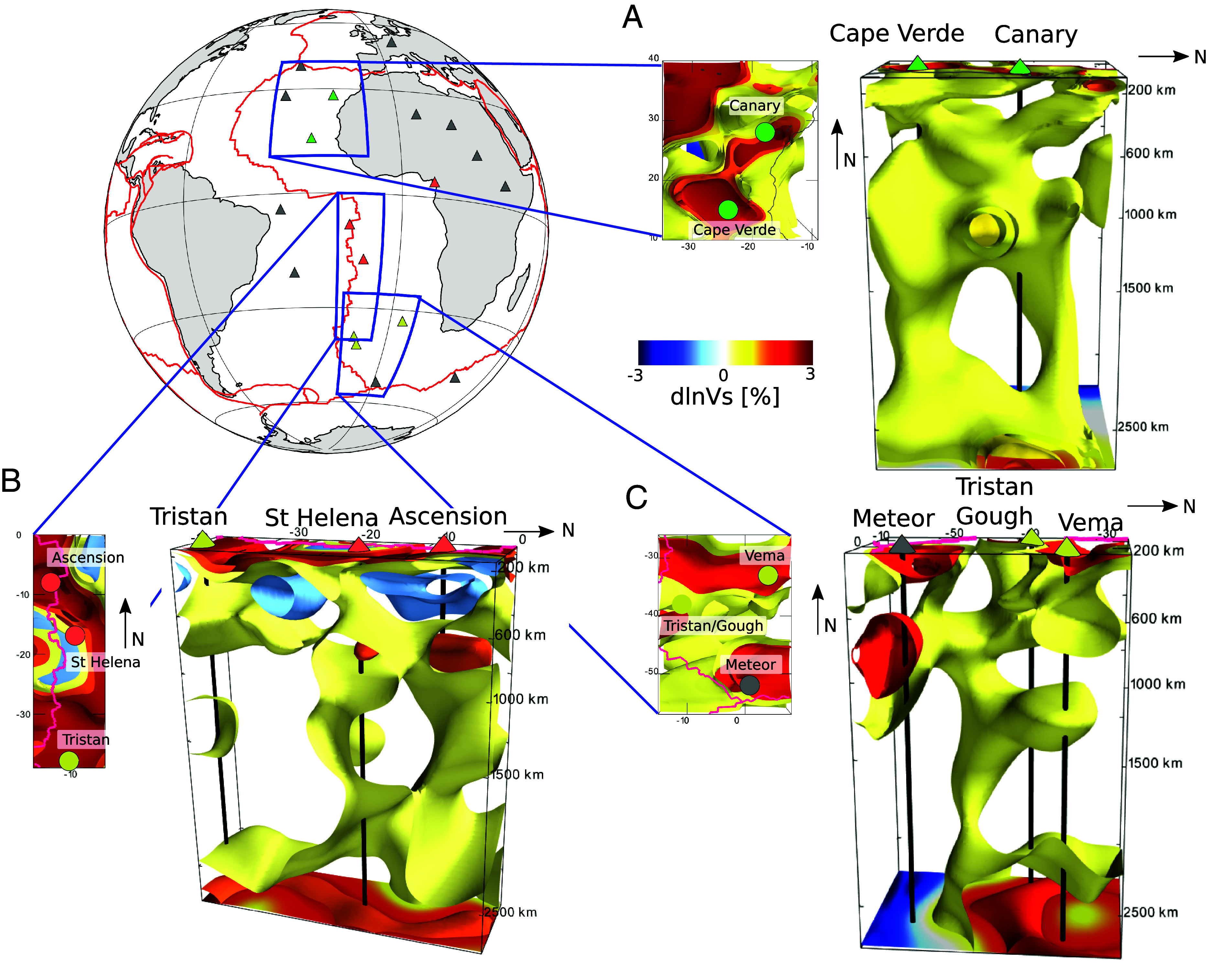
3D renders of shear velocity perturbations with respect to the 1D regional average (dlnVs, in percent) in model SEMATL_23 highlighting the following plume groups which are well separated from the core–mantle boundary to 1,000 km depth: (*A*) Cape Verde/Canary, (*B*) Ascension/St Helena, and (*C*) Tristan/Gough/Meteor/Vema. Black lines indicate vertical projection of the hotspot location down to the core–mantle boundary. *Top Left* map depicts the geographic extent (blue boxes) of each 3D render, with triangles indicating the location of major hotspots color-coded based on their similarity in isotopic signatures (Fig. 2). Zoom-in maps next to each render show dlnVs at 100 km depth.

Channel #1 lies above the Cape Verde/Canary plume group, which appears as a single low-velocity conduit in the lower mantle in some cross-sections (Fig. 2*A*), but at least partially separated into two conduits in the mid-mantle in others (Fig. 2*B*). This plume group is clearly distinct from that which is present beneath Ascension and St Helena (Fig. 2*B* and *SI Appendix*, Fig. S7), which, in turn, is located beneath the southwestern end of LVC #2. Both plume groups present significant horizontal deflection, at different depths in the extended transition zone (ETZ). Finally, the Tristan/Gough/Vema plume group beneath the southwestern end of LVC #3 is well separated from Ascension/St Helena across the whole lower mantle (Fig. 2*C* and *SI Appendix*, Fig S7), with a single conduit (or multiple ones, but unresolved) in the mid-lower mantle, deflected horizontally around 1,000 km depth,

Three-dimensional (3D) views of the model show the change of pattern between 600 and 1,000 km depth more clearly (Fig. 3). In the case of Canary/Cape Verde, two distinct conduits extend from the same or nearby patches at the CMB up to ∼1,200 km depth (Fig. 3*A*), where they appear to merge. They separate again around 500 km depth, one toward the southwest, the other toward the northeast, finally connecting to LVC #1 around 200 km depth. While the LVC broadens in the vicinity of each hotspot, the view from the top (map view) clearly shows the continuity of the LVC between the hotspots. On the other hand, resolution tests indicate that the separation and merging of the two conduits in the upper mantle and the mid-lower mantle is robust (*SI Appendix*, Fig. S7), but that we cannot yet distinguish between a single or distinct roots for these two plumes near the CMB.

Similarly to plume group #1, the 3D rendering for St Helena/Ascension (Fig. 3*B*) shows two separate conduits in the mid-lower mantle, merging around 1,000 km depth in a horizontally elongated zone, and separating again in the transition zone into two thinner more vertical conduits that eventually reconnect in LVC #2, in the vicinity of Ascension and St Helena, respectively. Here again, we cannot resolve whether these two conduits originate from a single patch or two separate ones at the CMB, but the latter is/are distinct from that/those of plume group #1 (e.g., Fig. 2*B*), as also shown in resolution tests (*SI Appendix*, Fig. S7). Finally, Fig. 3*C* shows what appears to be a single plume stem in the lower mantle beneath Tristan, branching out horizontally in different directions between ∼1,000 and ∼600 km depth

The ∼1,800 to 2,000 km horizontal spacing between the LVCs, as also seen in the Pacific Ocean basin (5, 18), combined with the horizontal deflection of mantle plumes around 1,000 km depth, as shown here and in other studies (e.g., refs. 3, 19, and 20) and evidence for the deflection of some slabs around the same depth (e.g., ref. 21) suggests the presence of mesoscale convection with an aspect ratio of roughly 1:1 in the top 1,000 km of the mantle, possibly associated with an increase in viscosity in the extended transition zone (e.g., ref. 22). We note that the presence of secondary scale convection in the Atlantic uppermost mantle has been suggested by Ma and Dalton (23) on the basis of correlations of seismic velocities and axial ridge depth anomalies, however in their model, the secondary scale convection is driven entirely from above by the cooling of the oceanic plate.

## Relation to Isotope Geochemistry

Interestingly, the three separate plume groups corresponding to LVCs #1 to #3 also correspond to three distinct signatures in radiogenic isotope space (e.g., Fig. 2 *D* and *E*). In isotope geochemistry studies, these known signatures are sometimes referred to, respectively, as LOND (Cape Verde/Canary), HIMU (Ascension/St Helena), and EMI (Tristan/Gough) and attributed to distinct sources in the deep mantle (24). It is often argued that HIMU reflects the sampling by deep mantle plumes of an old deep mantle reservoir of subducted oceanic crust while EMI holds the signature of delaminated subcontinental lithosphere (25) or upper mantle transition zone composition (26). However, the geochemical arguments have so far only found tenuous support from structural imaging.

The relation of isotope geochemistry and whole-mantle FWI tomography presented here allows us to refine our understanding of flow pathways from the core–mantle boundary to the surface, as expressed in intraplate volcanism, and as originally suggested by Castillo (27) and investigated in several global studies (e.g., refs. 28 and 29).

The tomographic modeling shows that the three plume groups correspond to distinct patches at the CMB (Fig. 3) which are well resolved (*SI Appendix*, Figs. S7–S9) and the plume groups are characterized by distinct isotopic signatures (Fig. 2 *D* and *E*). Thus, we interpret the geochemical variability between plume groups to reflect regional compositional heterogeneity in the lowermost mantle at the scale of 1,000 km or less, rather than differences in composition between the interior of the African LLSVPs and the deep ambient mantle (30, 31), or the consequence of vertical and lateral zonation within the LLSVP (e.g., refs. 31 and 32). Given our observations that the plumes merge, and then separate again in the upper mantle transition zone (more precisely the extended transition zone 400 to 1,000 km; Fig. 3), possibly indicating the existence of ponding zones in this region, the geochemical variability within each plume group might be acquired because of this more complex, and previously unrecognized, flow paths in the upper mantle and asthenosphere.

**Fig. 4.**
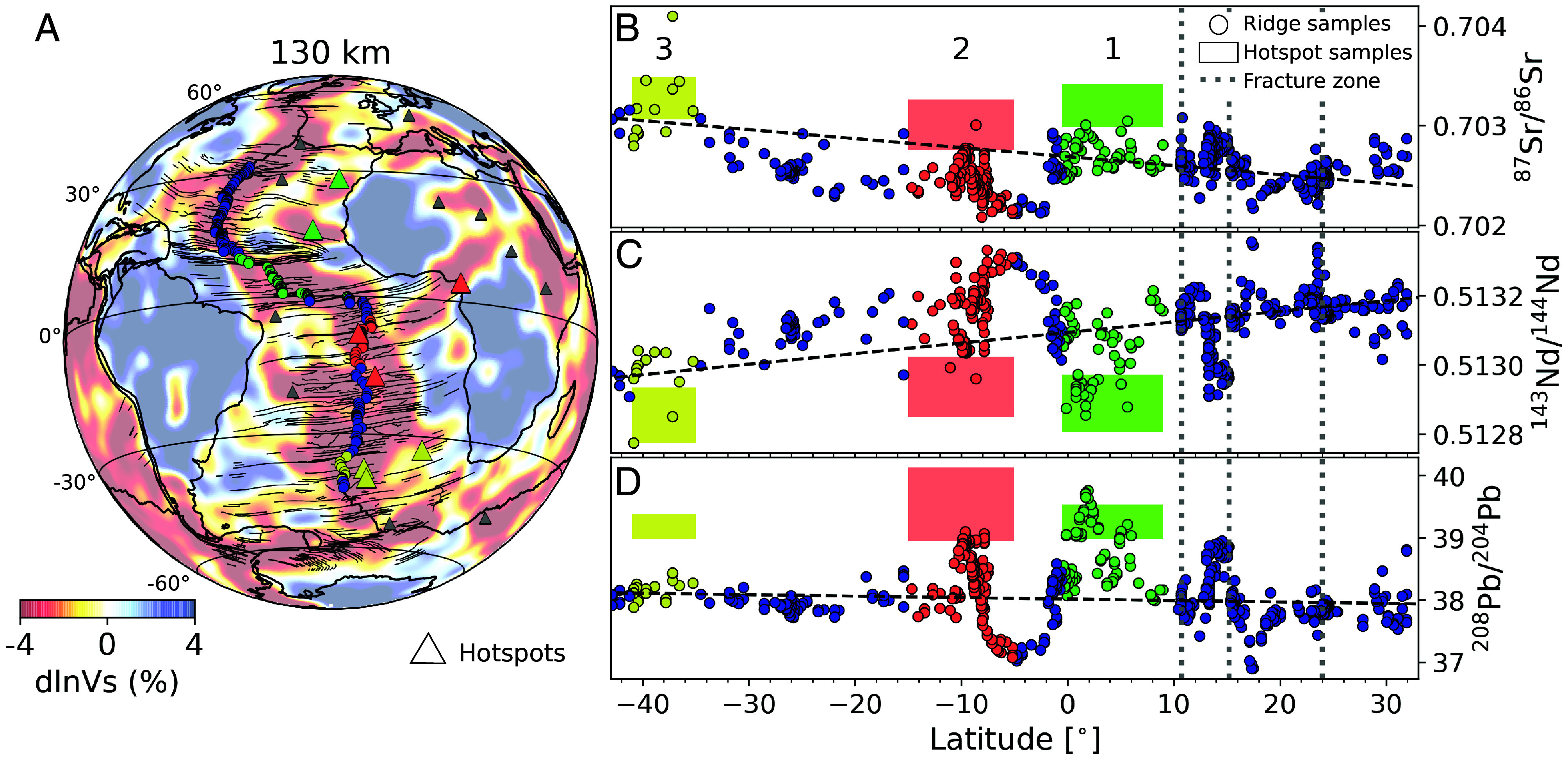
Radiogenic isotopic composition in MORBs along the MAR. (*A*) Map of relative variations in shear-wave velocity (dlnVs, in percent) at a depth of 130 km in model SEMATL_23. Black lines indicate major fracture zones and triangles denote major hotspots (7) in the study area. (*B*–*D*) Radiogenic isotopic composition of (*B*) ^87^Sr/^86^Sr, (*C*) ^143^Nd/^144^Nd and (*D*) ^208^Pb/^204^Pb in MORBs along the MAR (circles). The data were downloaded from the PetDB Database (www.earthchem.org/petdb) (33) on March 9, 2024. The latitude range of intersections of the LVCs with the MAR are indicated by different colors (yellow, red, and green). Mean values and SD of the corresponding isotopic anomalies (colored boxes) recorded at the respective major hotspots (Cape Verde/Canary for LVC#1; Ascension/St Helena for LVC#2; and Tristan/Gough for LVC#3) are placed for reference at the latitude of these intersections (as opposed to the actual latitudes of the hotspots, which, for Cape Verde/Canary, is particularly distinct). Broken lines indicate the average linear trend of anomalies (only for visual reference) for the blue samples, showing a slight latitudinal trend as noted by Stracke et al. (34). Vertical dotted lines indicate the latitude of major fracture zones (from left to right: Vema, 1520, and Kane fracture zones) on the African side of the MAR, oriented in an approximately east-west direction (different from the African APM and from the trend of the LVCs). Note in particular that the isotopic anomaly of the 1520 fracture zone (at a latitude close to that of Cape Verde) is weaker than that found at the intersection of the MAR with LVC #1, especially in ^206^Pb/^204^Pb. On the other hand, in the latitude segments corresponding to the intersection of the 3 LVCs with the MAR, the range of isotopic anomalies observed at the MAR match those of the corresponding hotspots for at least two of the three isotope pairs.

The consideration of distinct plume groups associated with upper mantle LVCs also brings a perspective on the interpretation of significant excursions from average mid-ocean ridge basalt (MORB) isotopic signature observed along the MAR. Such excursions have been explained as expressing the influence of near-ridge plumes on MORB (34–36), conducted along fracture zones. We propose, instead, that such excursions occur at the intersections of the ridge with the LVCs (Fig. 4), rather than at the latitude of the corresponding hotspots. This is particularly clear in the case of LVC #1, for which the strongest isotopic signature of the Cape Verde/Canary hotspot group occurs on the MAR in the latitude range of this intersection, rather than in the latitude range of these hotspots, which is $15^{{}^{\circ}}$ further north.

## Origin of the CVL

**Fig. 5.**
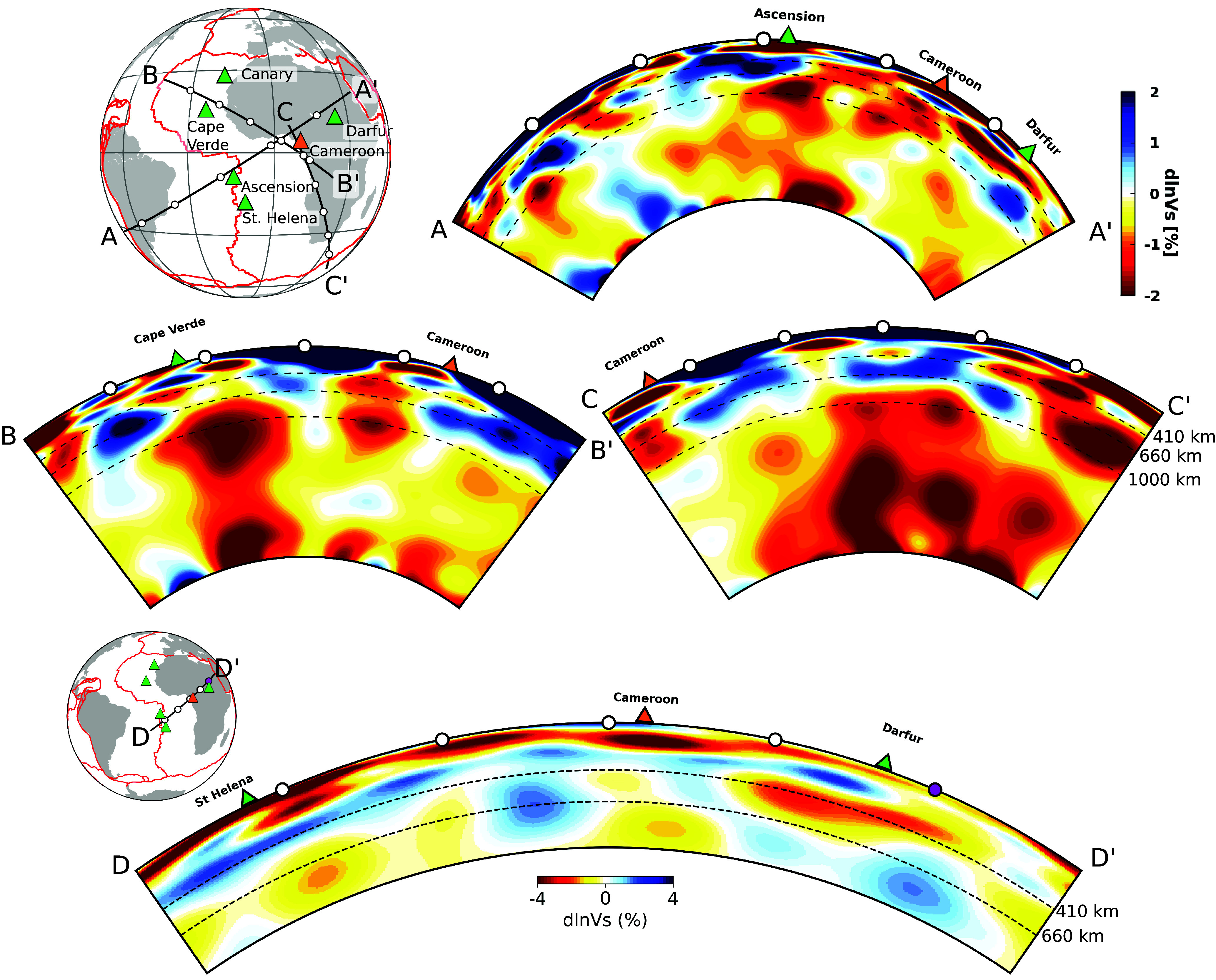
Whole-mantle depth cross-sections showing shear velocity perturbations (dlnVs, in percent) in model SEMATL_23 in the vicinity of the CVL. Red lines depict tectonic plate boundaries and colored triangles indicate major hotspots in the vicinity of Cameroon. (*A*–*A*′) Cross-section along a line linking the Ascension/St Helena plume group to Cameroon, showing a possible path-way linking these plumes to the Cameroon line. (*B*–*B*′) Cross section through the Cape Verde plume, showing no direct connection with the Cameroon line. (*C*–*C*′) This cross-section shows a group of strong plumes beneath south Africa that do not appear to connect to Cameroon in either the lower or the upper mantle. (*D*–*D*′) Zoomed in cross-section through the CVL, further hinting at the link between Ascension/St Helena plume group and the CVL.

Different interpretations have been proposed for the complex geochemical data (Fig. 2 *D* and *E*) from the CVL, located along LVC #2 (Fig. 1), with evidence for a link to an upper mantle upwelling, possibly a mantle plume (37, 38), contaminated to various degrees by oceanic and continental crust (39). However, there has not been conclusive evidence from seismology on the presence of any continuous low-velocity anomaly in the deep mantle beneath this mid-plate volcanic system. Some authors interpreted this in terms of edge-driven small-scale convection at the edge of the African continent (e.g., refs. 40, 41, 42). Celli et al. (13) proposed that the cratonic lithosphere between the west-African craton and the Congo craton has been eroded by the action of a mantle plume, but their model did not extend far enough in depth to identify the latter. It has been suggested that the Cameroon line may be connected to the northern edge of the African LLSVP (43), however, our model confirms the absence of a deep lower mantle plume directly beneath the Cameroon line (Fig. 5) and rules out a direct relationship with the African LLSVP, as the bundle of plumes rising from the center of the African LLSVP base does not extend north of latitude 10°S (Fig. 5*C*–*C*′). Instead, our model indicates a possible relation to the Ascension and St Helena plume group, as originally suggested by Morgan (14): a possible low-velocity conduit may exist starting from the extended transition zone (410 to 1,000 km) beneath Ascension/St Helena (Fig. 5*A*–*A*′), and merging into LVC #2 in the uppermost mantle. The upper mantle cross-section along LVC #2 (Fig. 5*D*–*D*′) shows the continuity of this LVC extending from St Helena to beyond the oceanic part of the Cameroon line, into the African continent.

## Discussion

Considering that the African plate motion is very slow (i.e., less than 500 km in the past 100 Myr; 44, 45), the relatively shallow expression of the LVCs, confined mostly within the seismic low-velocity zone above ∼200 km depth, but oriented roughly in the present APM direction, and occurring beneath the young portions of the oceanic plate, suggests the channeling of pressure driven asthenospheric flow from the southwest to the northeast, rather than small scale convection generated by the cooling of the plate, observed beneath old oceanic lithosphere (e.g., ref. 46), and as found in global modeling of interaction of plumes with plates (e.g., ref. 47). The quasi-periodic spacing and orientation of the LVCs, on both sides of the MAR, and the deeper upper mantle structure as highlighted in Fig. 3, suggests that another dynamic component needs to be considered: the plume flow in the southern and central Atlantic is not only influenced by plate motion but also interacts with an organized mesoscale convection system spanning across the depth of the extended transition zone. The ensemble of observations presented here helps bring together the results of numerous regional studies that have focused on one or the other of the volcanic lines associated with LVCs #1, #2, or #3.

We discussed LVC#2 and the Cameroon line in the previous section. Concerning LVC #1, by comparing early, lower resolution, seismic tomographic models with isotope geochemistry of cenozoic lavas, Hoernle et al. (48) suggested the presence of a 2,500 km long sheet-like upwelling in the upper mantle, extending from the Canary islands in the south-west to the western Mediterranean and the Rhine-Graben in the north-West (here, LVC #1). They suggested that this could help explain extensional tectonics in the western Mediterranean in spite of the dominant compressive regime at the African/European plate boundary (e.g., ref. 49). Further isotope analysis of lavas from the Canary hotspot track and northwest Africa, combined with evidence for thinned subcontinental lithosphere (e.g., ref. 50) suggested horizontal flow in the upper mantle over long distances, originating from the central Atlantic plume head (38), beneath the current location of the Cape Verde/western Canary Islands, and extending toward the northwest, all the way to the western Mediterranean (e.g., refs. 51 and 52). Our model brings out the deep mantle connection between LVC#1 and the Cape Verde/Canary hotspot group.

On the other hand, LVC #3 lies beneath the long hotspot and seamount track that extends from the presently active Tristan da Cuhna and Gough hotspots, along the Walvis ridge, all the way to the Etendeka flood basalt province. The geological and geochemical connection along this volcanic line is well documented (e.g., ref. 53). Notably, it has been related to a plume source at the base of the mantle based on its DUPAL geochemical signature (54). Here also, our model brings out the corresponding deep mantle plumbing.

Interestingly, Meyers et al. (55) noted the similarity of geological features in the volcanic and seamount chains extending in a quasi-parallel way, SW to NE, with separations of ∼1,700 km, across the western side of Africa, providing evidence for uplift of pre-Miocene crust and sediments, and contemporaneous volcanism since the Miocene, often without consistent age progression. Meyers et al. (55) called them “hot lines”—following Bonatti et al. (56)—and suggested they are manifestations of linear upwelling zones of hot mantle, as would manifest the presence of secondary scale convective rolls in the upper mantle, coupled to the lithosphere. Unlike the classical “Richter rolls” (57), which are formed as a result of shear at the lithosphere-asthenosphere boundary, Meyers et al. (55) proposed a possible role for unidirectional shear at the base of this secondary scale convection layer, which, they speculated, could be located at the 670 km discontinuity. While their interpretation was highly speculative and based primarily on shallow seismic reflection profiles, stratigraphic relationships, and the petrology and geochronology of lavas, the tomographic and geodetic observations presented here support this type of model and provide further constraints on the depth range of the corresponding upwellings.

## Conclusions and Outlook

Our study shows the presence of separate, vertically oriented plume groups rooted at the core–mantle boundary, each associated with an LVC in the uppermost mantle, elongated in the direction of present APM and carrying a specific isotope geochemical signature, at least in the Sr/Pb/Nd space. The corresponding deep mantle upwelling flow interacts with secondary scale convection in the extended transition zone that is at least partially driven from below, and not only by the motion of the plate above. Our modeling also confirms the horizontal deflection of the implied upwelling flow around 1,000 km depth, sometimes involving spreading for long distances in the extended transition zone and/or upper mantle, before being expressed in volcanism at the surface, as has been observed for many other deep mantle plumes around the world (e.g., refs. 3, 19, 20, 58, and 59). Spreading of low-velocity material from beneath southern Africa (e.g., ref. 60) toward the north-east has also been suggested to connect to the observed volcanism beneath the East African Rift. This change of pattern in the top 1,000 km of the mantle, the merging of separate columns within a given plume group, and their subsequent separation in the extended transition zone (Fig. 3), taken together with the 2,000 km separation between the LVCs in the asthenosphere (Fig. 1), supports a model of interaction of deep mantle plumes with secondary scale convection of aspect ratio close to 1:1 in the top ∼1,000 km of the mantle.

The most puzzling and intriguing aspect of this work is the complementary, interacting roles of lower mantle upwellings and upper mantle mesoscale convection in shaping the seismic structure of the upper mantle, the geochemistry of intraplate lavas, and the age progression of volcanism. The quasi-periodic spacing between LVCs suggests a dynamical control, but it remains unclear whether this periodicity is set by the natural organization of deep mantle plumes or by a preferred wavelength of upper mantle small-scale convection, controlled by viscosity layering and plate motion. Reconciling the LVC observations presented here for the south-Atlantic region with those in the central Pacific ocean, which have similar spacing, but a deeper signature and a different relation with geodetic observables (18) should shed further light on the underlying dynamics. Investigations of the interaction between large-scale flow and mesoscale convection in the upper mantle have so far been few/limited beyond the specific interaction of plate motion and simplified plume models (7). Further geodynamical modeling allowing significantly distinct rheologies in the top third and bottom two-thirds of the mantle combined with seismological studies focused on the extended transition zone are needed to further advance our understanding of these processes. There is also room for improvement of the tomographic resolution to further confirm the continuity and better characterization of the LVCs on both sides of the MAR.

Finally, as tomographic resolution improves, it becomes progressively clearer that the LLSVPs are not topologically compact (unbroken), dense piles extending high across the lower mantle, but rather a bundle of individual mantle plumes, well separated from each other at their very base (20, 61). Some holes have been previously reported in the African LLSVP (20, 61, 62). Model SEMATL_23 confirms the latter is broken up into separate thermo-chemical plumes, with roots tapping distinct isotopic reservoirs.

## Methods

Building up on previous work (e.g., refs. 6 and 63), we here implemented a hybrid full-waveform algorithm that combines the accuracy of the Spectral Element Method (SPECFEM3D_Globe; 64, 65) for the forward simulation of seismic waveforms, with a Gauss–Newton quadratically converging inversion scheme, in which we compute the approximate Hessian using normal mode perturbation theory (NACT; 66). This approach allows us to construct the full physics-based approximate Hessian matrix, and employ a rapidly converging Gauss–Newton optimization scheme, ultimately decreasing the computational cost of the inversion by reducing the number of forward wavefield simulations required.

In order to enhance the model resolution over the target area (dashed green line in Fig. 1), we compiled a dataset of three-component acceleration seismograms recorded at permanent and temporary broad-band seismic stations from 301 events ensuring, inasmuch as possible, uniform coverage over the central and southern Atlantic ocean (latitude: 58°S–36°N and longitude: 83°W–43°E). All seismic data were downloaded from the EarthScope Consortium Web Services (https://service.iris.edu/) through the Python toolbox ObsPy ( 67, 68, 69). Starting from SEMUCB_WM1 as initial model, 3-D variations in Voigt-average isotropic shear-wave velocity ($V_{\;\text{s}}$) and radially anisotropic parameter ($\xi =V_{\;\text{SH}}^{2}/V_{\;\text{SV}}^{2}$) in the mantle are updated using a two-step approach successively for upper mantle and lower mantle structure. Further details are given in *SI Appendix*, section 1.

## Supplementary Material

Appendix 01 (PDF)

## Data Availability

Model SEMATL_23 data have been deposited in IRIS Earth Model Collaboration (https://ds.iris.edu/ds/products/emc/) (69).
