# Supplementary material for: Deep mantle plumes feeding periodic alignments of asthenospheric fingers beneath the central and southern Atlantic Ocean
Source: Proc Natl Acad Sci U S A. 2024 Nov 5;121(46):e2407543121. doi: 10.1073/pnas.2407543121 (PMC11573586; doi:10.1073/pnas.2407543121)
Supplement: Supplementary file 1 — Appendix 01 (PDF) [file pnas.2407543121.sapp.pdf]

Supporting Information for

Deep mantle plumes feeding periodic alignments of asthenospheric fingers beneath the central and southern Atlantic Ocean

Federico D. Munch, Barbara Romanowicz, Sujoy Mukhopadhyay, and Maxwell L. Rudolph

Barbara Romanowicz.
E-mail: barbararberkeley.edu

This PDF file includes:

Supporting text
Figs. S1 to S10
Table S1
SI References

Supporting Information Text

Methods. Building up on previous work by the Berkeley group (1, 2), we here implement a hybrid full-waveform algorithm that combines the accuracy of the Spectral Element Method (SPECFEM3D_Globe; 3–5) for the forward simulation of seismic waveforms, with a Gauss-Newton quadratically converging inversion scheme, in which we compute the approximate Hessian using normal mode perturbation theory (NACT; 6). The use of the NACT approximation allows us to compute 2D Fréchet derivatives in the vertical plane containing the source and the receiver, which better reflects the sensitivity of body waves and overtones to mantle structure than the frequently used "path average" approximation (PAVA; 7, 8), in which the Fréchet derivatives vary only in depth and are strictly valid only for isolated mode branches (i.e. fundamental mode surface waves). Note that these Fréchet derivatives are updated at each iteration, accounting for the evolution of the average 1D model on each source station path. The Hessian matrix is then constructed from these 2-D Fréchet derivatives by combining tens of thousands of paths (for further details, the reader is referred to 6). This approach allows us to construct the full physics-based approximate Hessian matrix, and employ a rapidly converging Gauss–Newton optimization scheme, ultimately decreasing the computational cost of the inversion by reducing the number of SEM-based forward wavefield simulations required.

Within this framework, the mantle structure is updated using a two-step approach. First, we invert seismic waveforms in the period range 40-300 seconds (i.e., datasets A and B described in the Data Section broken up into wavepackets containing fundamental mode and overtone surface waves) to retrieve variations in isotropic shear-wave velocity (V_s) and radial anisotropy ($\xi = V_{\mathrm{SH}}^2/V_{\mathrm{SV}}^2$) from 80-km depth down to 800-km depth. After three iterations, we observe no significant changes in the overall misfit and, subsequently, the retrieved mantle structure. In a second step, we fix the 3D structure obtained in the first 400 km of the mantle, and perform three additional iterations in which we invert seismic waveforms in the period range 30-300 seconds (i.e., datasets A and C in Section , broken up into wavepackets containing different body wave phases) to retrieve changes in V_s and ξ between 400-km depth and the core-mantle boundary (∼2900 km depth). The variance reduction for the initial and final models of the surface wave and bodywave inversions, respectively, is presented in Table S1. At each iteration, a model update is calculated as

$$\mathbf{m}_{k+1} = \mathbf{m}_k + (\mathbf{C}_m\mathbf{G}^T\mathbf{C}_d^{-1}\mathbf{G} + \mathbf{I})^{-1} \times (\mathbf{C}_m\mathbf{G}^T\mathbf{C}_d^{-1}[\mathbf{d} - g(\mathbf{m}_k)] + \mathbf{m}_p - \mathbf{m}_k], \qquad [1]$$

where $\mathbf{m}_k$ is the current model (i.e., recovered at the *kth* iteration), $\mathbf{m}_p$ is the a-priori model (i.e., *SEMUCB_WM1*), the vectors $\mathbf{d}$ and $g(\mathbf{m}_k)$ represent the observed and modelled waveforms, while $\mathbf{G}^T\mathbf{C}_d^{-1}\mathbf{G}$ is the NACT-based Hessian. The data covariance matrix $\mathbf{C}_d$ is a diagonal matrix with entries that reflect data uniqueness and quality (see Appendix A in 9). Finally, the model covariance matrix $\mathbf{C}_m$ limits the acceptable length-scales of heterogeneity within the model. This matrix is based upon prescribed model-parameter variances and correlation lengths, with the latter spatially tuned according to data-coverage density and quality. As in our previous work (2), the elements of $\mathbf{C}_m$ jointly describe the state of a-priori information on model coefficients i and j as follows:

$$(\mathbf{C}_m)_{ij} = \sigma_0\sigma_{ij}\exp\left[\frac{cos\Delta_{ij} - 1}{h_{ij}^2}\right]\exp\left[-\frac{r_{ij}}{v_{ij}^2}\right], \qquad [2]$$

where Δ_{ij} and r_{ij} are the minor-arc distance and radial separation between i and j, respectively. The quantities v_{ij} and h_{ij} are the radial and lateral correlation lengths, with h_{ij} being normalized to the range $0 \leq |\cos\Delta_{ij}1| \leq 1$. $\sigma 0 isaconstant, "norm - damping" factor.Finally,$$\sigma_{ij}$ is a constant near-unit variance pre-factor assigned to each of V_s and ξ.

Following our previous work, we prescribe radial correlation lengths with a fixed depth dependence ranging from 50 km at the shallowest upper-mantle depths up to 300 km in the mid-mantle. Moreover, we implement an adaptive lateral correlation-length scheme based on the diagonal entries of the Hessian to represent aggregate sensitivity of the data to each model coefficient. Here, contributions from different data are weighted according to the quality and uncertainty estimates appearing in $\mathbf{C}_d$. These values are, in turn, used to select 'local' correlation-length estimates by scaling to the interval between prescribed minimum and maximum values for a given model parameter - set to 400–1200 km for both V_s and ξ.

Model parameterization. Building up on our previous work (1, 2), we here invert for 3-D variations in Voigt-average isotropic shear-wave velocity V_s and the radially anisotropic parameter $\xi = V_\mathrm{SH}^2/V_\mathrm{SV}^2$ in the mantle, with respect to a 1-D reference model that evolves throughout the inversion by removing the degree-0 component from the 3-D structure at each iteration. Variations in the remaining four parameters of a radially anisotropic medium V_p, $\Phi = V_\mathrm{pv}/V_\mathrm{ph}$, η, and ρ (see definitions in 10) are scaled using empirical relationships (11). Perturbations to V_s and ξ inside the target area are expressed in terms of cubic b-splines $\nu_q(r)$ radially (e.g., 12) and in spherical splines $\beta_p(\theta, \phi)$ laterally (13). Hence, the value of a given model parameter m at any given location in the Earth (θ, ϕ, r) can be calculated from a set of spline coefficients m_{pq} as

$$m(\theta, \phi, r) = \sum_p \sum_q m_{pq} \beta_p(\theta, \phi) \nu_q(r). \quad [3]$$

We here adopt the same radial b-spline basis used in *SEMUCB_WM1* (i.e., 20 knots with variable spacing between the core-mantle boundary and 30-km depth), whereas the spacing of the knots associated with the spherical-spline basis are refined inside the target area (see Fig. S1a). The spherical knots have a lateral spacing $\approx 1°$ for V_s and $\approx 2°$ for ξ inside the target area resulting in 7974 and 2574 unknown model parameters in V_s and ξ at each radial knot depth. This represents a significant reduction in lateral spacing with respect to the initial model ($\approx 2°$ in V_s and $\approx 8°$ in ξ). As shown in Fig. S1b, radial splines are clustered more tightly near the top and bottom of the mantle model as we aim at capturing the stronger heterogeneity in the uppermost mantle and in D$''$.

The existence of thin layers with low seismic velocities within the Earth's crust significantly slows down the SEM wavefield calculations (14). In order to reduce the computational cost of our waveform simulations, we take into account the effect of the crustal structure on the seismic wavefield by resorting to an homogenization scheme (e.g., 15, 16). Such a scheme replaces a set of thin discontinuous layers by a single, smoothly varying anisotropic layer that has an equivalent seismic response at long periods (i.e., equivalent to the zeroth-order scheme of 17). As discussed extensively in previous work (2), this approach leads to no significant loss of accuracy for long-period waveform modelling while significantly reducing the computational cost of the waveforms simulations.

Similarly to *SEMUCB_WM1*, we build a homogeneized crustal model by fitting surface wave group-velocity dispersion data(18) in the period range 25-60 seconds, while fixing the Moho depth to CRUST2.0 model (19) when the thickness of the crust is larger than 30 km, fixing it to 30 km otherwise. Crustal shear-wave velocity and radial anisotropy structure are parametrized as a function of depth using degree-4 Lagrange polynomials with Gauss-Lobatto-Legendre interpolation points of the same type used in the SEM - a similar approach to that taken by Fichtner et al (20) in their alternative implementation of a smooth crustal layer.

Because of trade-offs between crustal structure and uppermost mantle structure, we also include the uppermost mantle, down to a depth of 150 km when updating the crust. We iteratively invert for crustal radially anisotropic structure, constrained by dispersion in the period range 20-60s, and uppermost mantle structure, constrained by dispersion from the same data collection in the period range 60 to 150 seconds, following the approach of Chen and Romanowicz (21). Updates in isotropic shear-wave velocity and radial anisotropy are computed by means of a Gauss-Newton method, using *SEMUCB_WM1* crustal and uppermost mantle model as initial model. Iterations are performed until no significant changes in the misfit nor velocity variations are observed - which is achieved after 15 iterations. Once converged, we fix the structure in the top 80 km in subsequent iterations for deeper mantle structure using full waveform inversion.

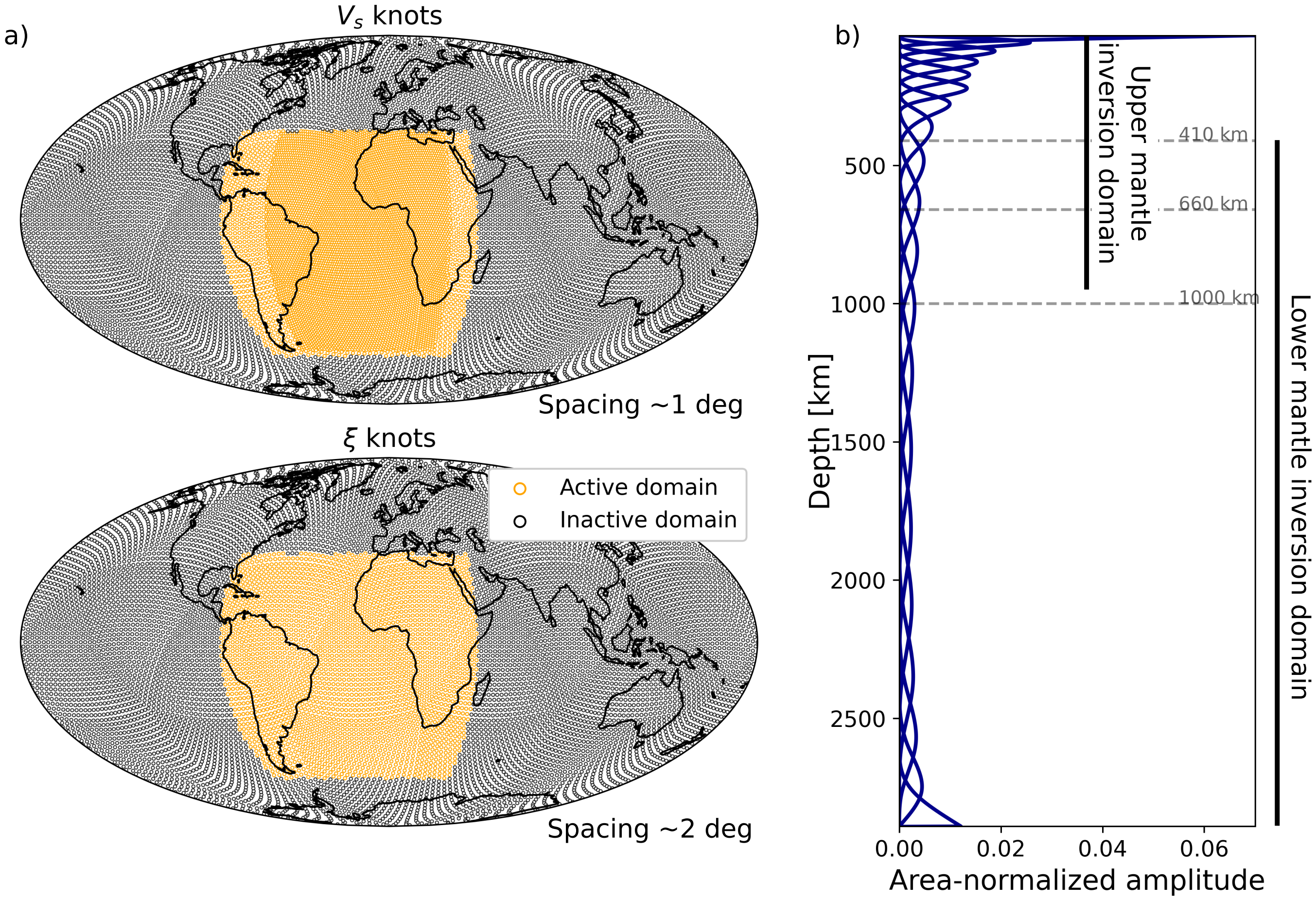

Fig. S1. Model parameterization considered in this study. (a) Lateral distribution of knots supporting the spherical splines (13) basis for isotropic shear-wave velocity (V_s) and radial anisotropy ($\xi = V_{\mathrm{sh}}^2/V_{\mathrm{sv}}^2$) with the active inversion domain indicated in orange. (b) 20 radial b-spline basis functions (12) distributed between the shallowest Moho depth in our homogenized crustal model and the core-mantle boundary. The locations of the knots are at the following depths: 30-km, 51-km, 81-km, 121-km, 171-km, 221-km, 271-km, 321-km, 471-km, 621-km, 796-km, 996-km, 1221-km, 1521-km, 1821-km, 2096-km, 2371-km, 2596-km, 2771-km, and 2891-km.

Data. The data considered in this study consists of: 1) three-component acceleration seismograms recorded at permanent and temporary broad-band seismic stations from 301 events ensuring, inasmuch as possible, uniform coverage over the central and southern Atlantic ocean; and 2) Love and Rayleigh waves group velocity maps in the period range 25 to 150 s (18). For the former, only events with moment magnitudes (M_w) larger than 4.5 were considered to guarantee a good signal-to-noise ratio and very large events (i.e., $M_w > 7.2$) were avoided to prevent possible complications associated to their long source-time duration, which makes the point source assumption invalid. Moreover, in order to avoid superposition of energy from different events, we ensured that all events satisfy the following criteria: 1) no $M_w > 7$ events reported up to 2 days before the origin time of the event (here-in-after, OTE); 2) no $M_w > 6$ events recorded one day before the OTE; and 3) no $M_w > 5$ events reported in the 6 hours prior to the OTE. Moreover, seismic traces within 15° of the source or the antipode were excluded from our data set, as the asymptotic coupling theory used in the calculation of the partial derivatives (describes in the Methods section) breaks down near $\Delta = 0°$ and $\Delta = 180°$ (22). Event hypocentre coordinates, moment tensor components, and origin times were taken from the Centroid Moment Tensor catalogue (http://www.globalcmt.org).

The observed waveforms were divided into three subsets (see Fig. S2):

1. Data set A: waveforms from 251 regional events ($4.5 < M_w < 6.5$) with sources and stations mainly located within the target region. The waveforms (with record length of 4,200 s) comprise minor-arc surface and body waves.

2. Data set B: waveforms (record length of 10,000 s) from 20 teleseismic events ($5.5 < M_w < 7.1$) with sources and stations distributed across the globe to capture second-orbit fundamental-mode Love and Rayleigh waves travelling across the target area.

3. Data set C: records with duration of 3,000 s from 30 teleseismic events ($5.5 < M_w < 7$) with sources and stations distributed globally to capture body waves (i.e., ScS, ScS_2, and S_{diff}) illuminating the mid- and lower mantle beneath the target region.

Datasets A and C span the time period 2001-2020 while Dataset C is a subset of the waveform dataset collected for the construction of global model *SEMUCB_WM1* (2). Building upon previous work (2), we incrementally incorporate higher frequency data as the inversion progresses by filtering the waveforms in two frequency bands: 1) 40 and 300 s, with corner frequencies at 53 and 180 s, respectively; and 2) 30 and 300 s, with corner frequencies at 36 and 180 s, respectively. We note that both frequency bands differ from the ones used in the construction of *SEMUCB_WM1* - for which cut-off periods were set to 60-400 s and 32-300 s, respectively. At each iteration of the inversion, observed and synthetic waveforms are compared using a two step procedure (see details in 9, 10) that makes use of both phase and amplitude information. The first step considers the entire trace to exclude: 1) noisy data; 2) seismograms with an incorrect instrument response; and 3) traces containing glitches. In the second step, the waveforms are then divided into wave packets or windows containing one or several known seismic phases, and labelled accordingly. Wavepackets are selected by comparison with the corresponding synthetics, using a set of criteria including variance reduction and cross-correlation (e.g., 23).

In the period range 40-300 s, wavepackets are separated according to whether they contain Love and Rayleigh 1st or 2nd orbit fundamental mode, overtone or mixed (i.e., overtone and fundamental mode) energy, whereas wavepackets in the period range 30-300 s are classified based on the travel-time of body wave phases (e.g., S, SS, SSS, ScS, ScS_2, and S_{diff}, among others). We note that a given body wavepacket may contain several body wave phases. Figs. S3 and S4 summarize the number and type of wavepackets picked for each frequency band. Finally, the wavepackets are weighted according to path redundancy, amplitude and data uncertainty and/or noise estimates (9, Appendix A).

Linear resolution analysis. In order to assess the robustness of the features present in our model, we here perform a series of standard resolution analyses. In such a framework, the resolution operator $\mathbf{R} = \mathbf{I} - \mathbf{C}'_{\mathbf{m}}\mathbf{C}_{\mathbf{m}}^{-1}$, with posterior covariance operator $\mathbf{C}'_{\mathbf{m}} = (\mathbf{G}^{\mathbf{T}}\mathbf{C}_{\mathbf{d}}^{-1}\mathbf{G} + \mathbf{C}_{\mathbf{m}}^{-1})^{-1}$, depends upon the data and model prior covariance operators ($\mathbf{C}_{\mathbf{d}}^{-1}$ and $\mathbf{C}_{\mathbf{m}}^{-1}$) as well as the model Jacobian $\mathbf{G}$. The resolution operator $\mathbf{R}$ can be viewed as a filter applied to a hypothetical model perturbation ($\mathbf{m}'$). The difference between $\mathbf{m}'$ and $\mathbf{Rm}'$ provides insights into what scales of solution structure might be interpretable and highlights pathological conditions such as strongly non-uniform data coverage (resulting in smearing). It is clear that if the data supplies no constraint on the model, then $\mathbf{C}_{\mathbf{m}} = \mathbf{C}'_{\mathbf{m}}$ (i.e., $\mathbf{R} = 0$) and, as a consequence, nothing is resolved. As an example of the multiple tests performed, Figs. S7-S9 illustrate the ability of our tomographic setup to image vertical plume-like conduits and compact structures extending from the core-mantle boundary into the mid-mantle. It must be mentioned that $\mathbf{R}$ only encapsulates the a-priori estimates of data noise and model uncertainty/smoothness, as well as spatial variation in data coverage characterized by $\mathbf{G}$. However, any reflection upon the accuracy of the modelling theory is conspicuously absent. In addition, the resolution operator is assembled in the last iteration leading up to model *SEMATL_23*, a 3D model which contains plume structures, resulting in interesting "memory" effects - such as the faint plume-like shadows in the mid-lower mantle shown in Fig. S9 - due to the fact that the partial derivatives that enter in the construction of the resolution operator are computed using NACT, which includes multiple-forward scattering in this 3D model, rather than being referenced to a 1D model. It is not possible to perform a resolution analysis referred to a 1D background model, without redoing the 5 iterations of the model from scratch, a limitation of this resolution analysis, which is still much more efficient than the required analysis of point-spread functions used in adjoint-based full waveform inversions (23).

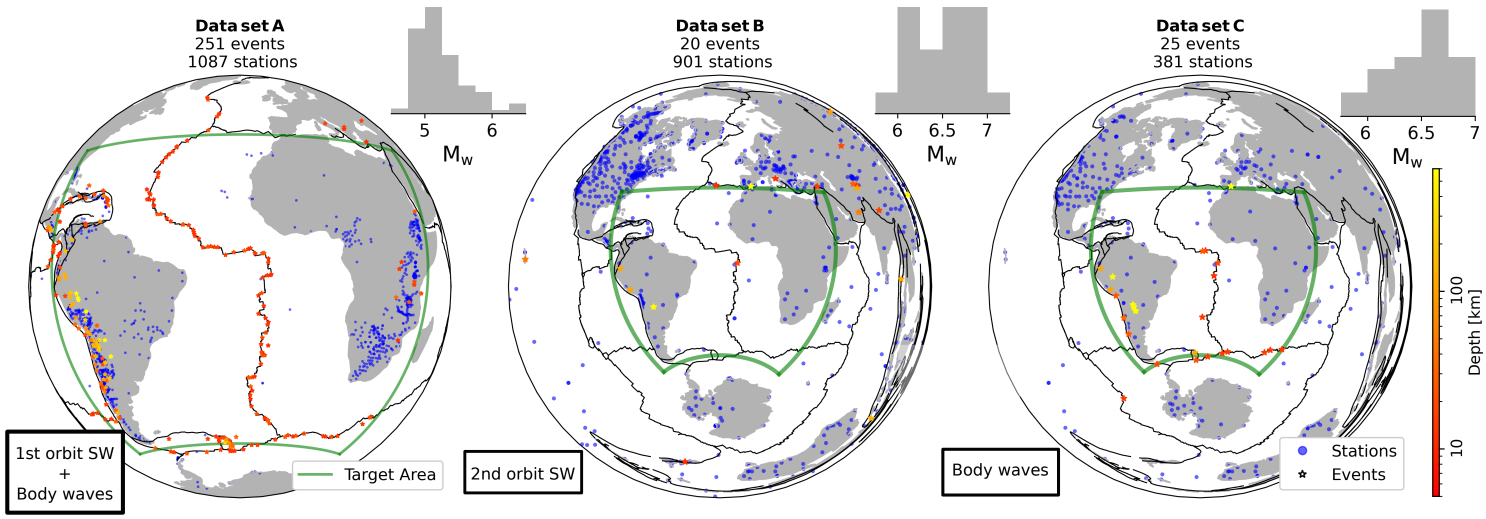

Fig. S2. Geographic distribution of seismic stations (blue circles) and events (stars color-coded by depth) for the three data sets considered in this study (see details in Data Section). Green solid line indicates the area of interest (i.e., inversion domain) and histograms summarize the range of moment magnitudes (M_w) contained in each data set.

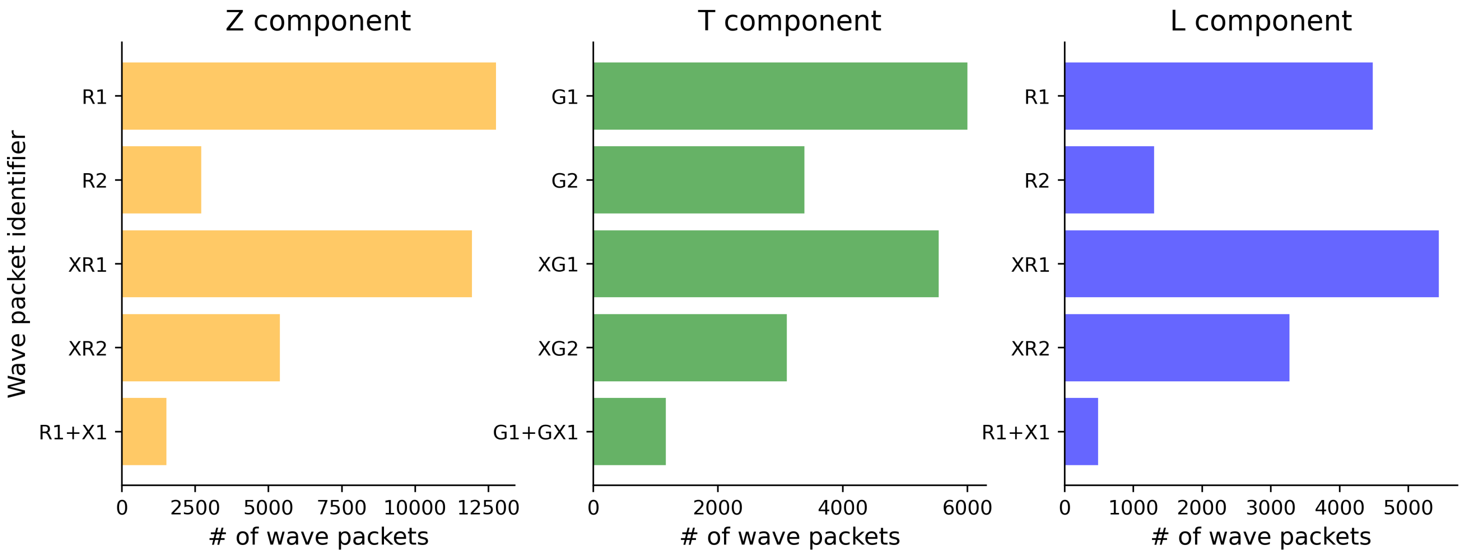

Fig. S3. Number and type of surface wave packets picked for each component in the period range 40-300 seconds. In the inversion, the wavepackets are weighted to avoid oversampling of certain paths (see details on the weighting scheme in (9)).

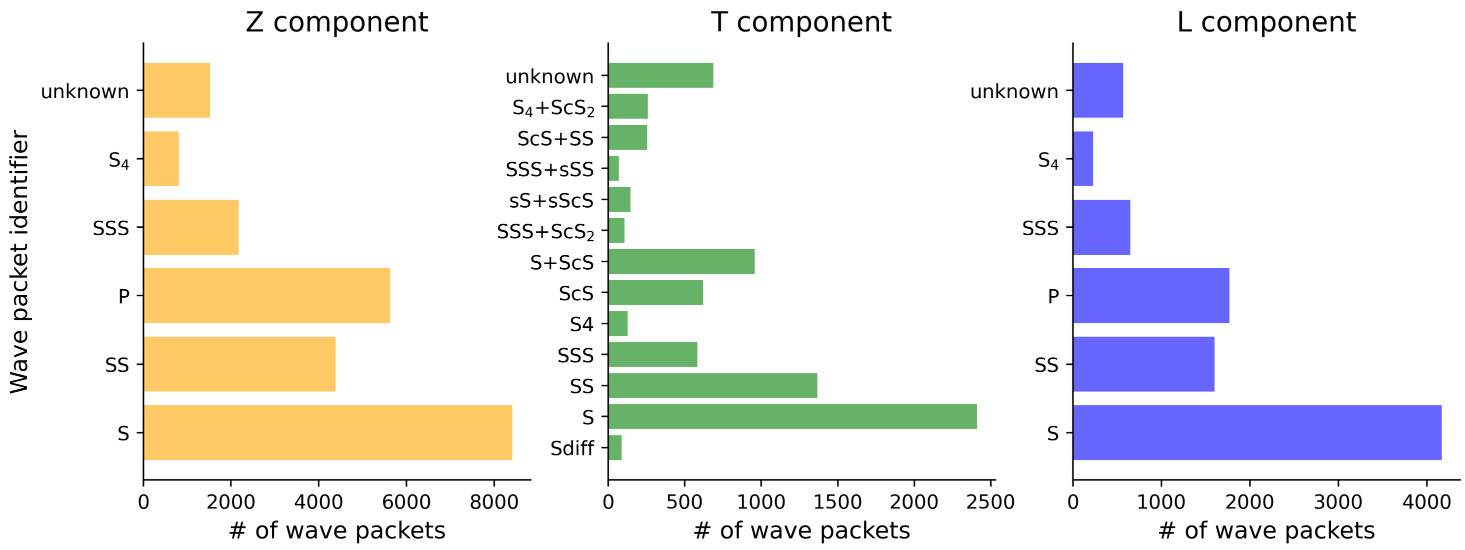

Fig. S4. Number and type of body wave packets picked for each component in the period range 30-300 seconds. The seismic phases included in these wave packets are indicated next to each bar. "Unknown" means that no specific body wave phase was associated with the corresponding wave-packets. Body wave packets are also weighted to limit oversampling of certain paths that depend on the source station distance and the seismic phase considered.

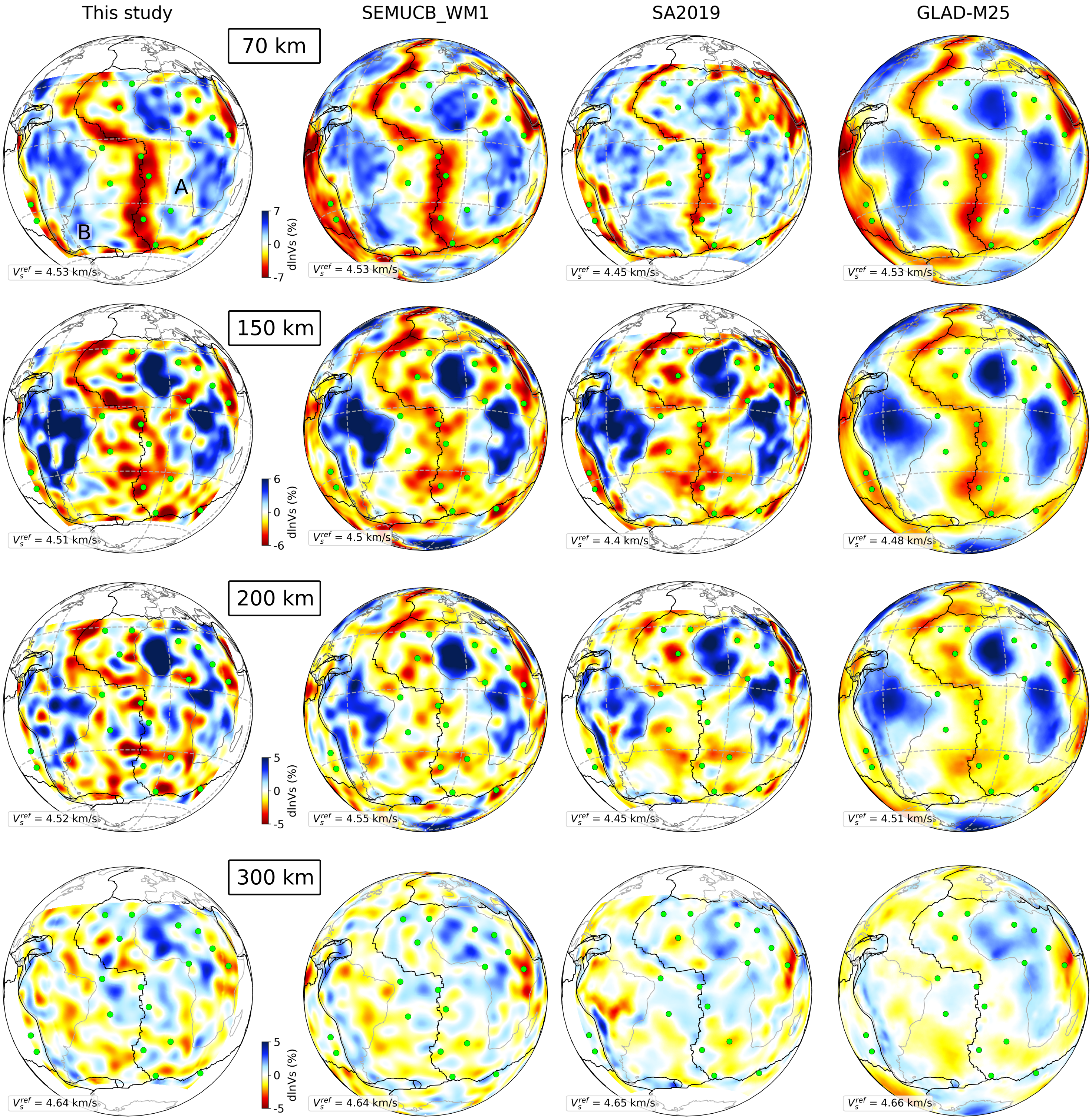

Fig. S5. Variations in isotropic shear velocity structure (V_S) in model *SEMATL_23*, compared to two other recent global models - *SEMUCB_WM1* (2) and *GLAD-M25* (24) – and the regional model *SA2019* (25) plotted in map view at several upper-mantle depths. Model structure is plotted as variations (%) with respect to the 1D average associated with each model ($\mathrm{V_s^{ref}}$) at the corresponding depth. Green circles denote location of major hotspots of (26) and solid lines indicate plate boundaries.

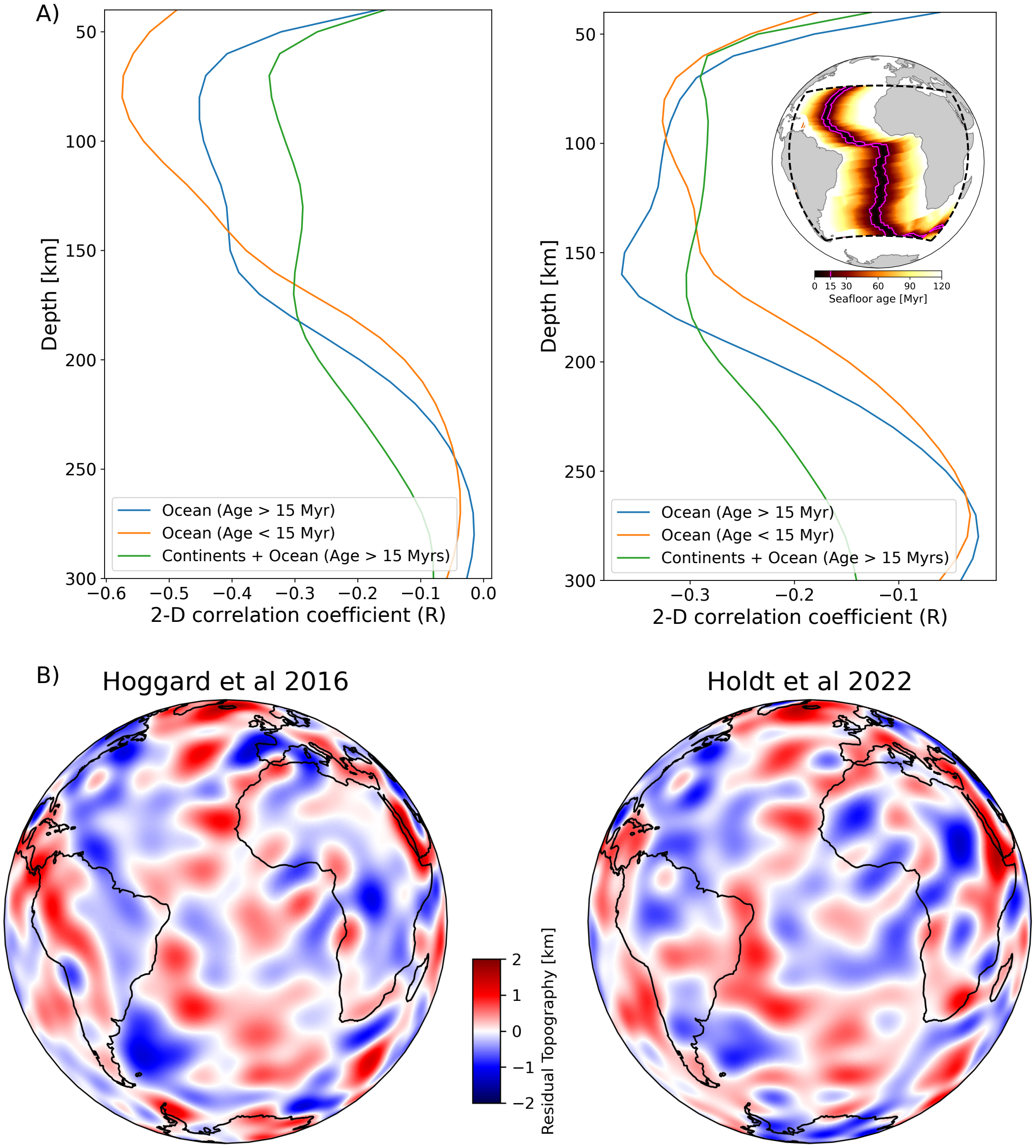

Fig. S6. (A) Correlation coefficient between the shear-wave velocity variations derived in this study and residual topography (i.e., proxy of dynamic topography filtered) between harmonic degrees 4 and 30 by (left) Hoggard et al (27) and Holdt et al (28) in the study area (black dashed lines in the inset map) as a function of depth. Correlation coefficients were computed for: 1) oceanic area restricted to the mid-ocean ridge (i.e., sea floor age $<$ 15 Myr, orange); 2) oceanic area excluding the mid-ocean ridge (i.e., for sea floor age $>$ 15 Myr, blue); and 3) combined continental and oceanic regions, but excluding the ridge (i.e. ocean sea-floor younger than $<$ 15 Myr, green). Inset map shows sea floor age taken from Seton et al (29) and magenta solid line indicates the 15 Myr age contour. We find larger (negative) correlations between the shear-wave velocity variations and the estimates by Hoggard et al. (B) Comparison between residual topography by (left) Hoggard et al (27) and Holdt et al (28) between harmonic degrees 4 and 30. The differences between the maps - specially in oceanic regions - illustrate the current uncertainties in the construction of residual topography estimates, particularly in the treatment (i.e., geodetic corrections) of regions in the vicinity of hotspots.

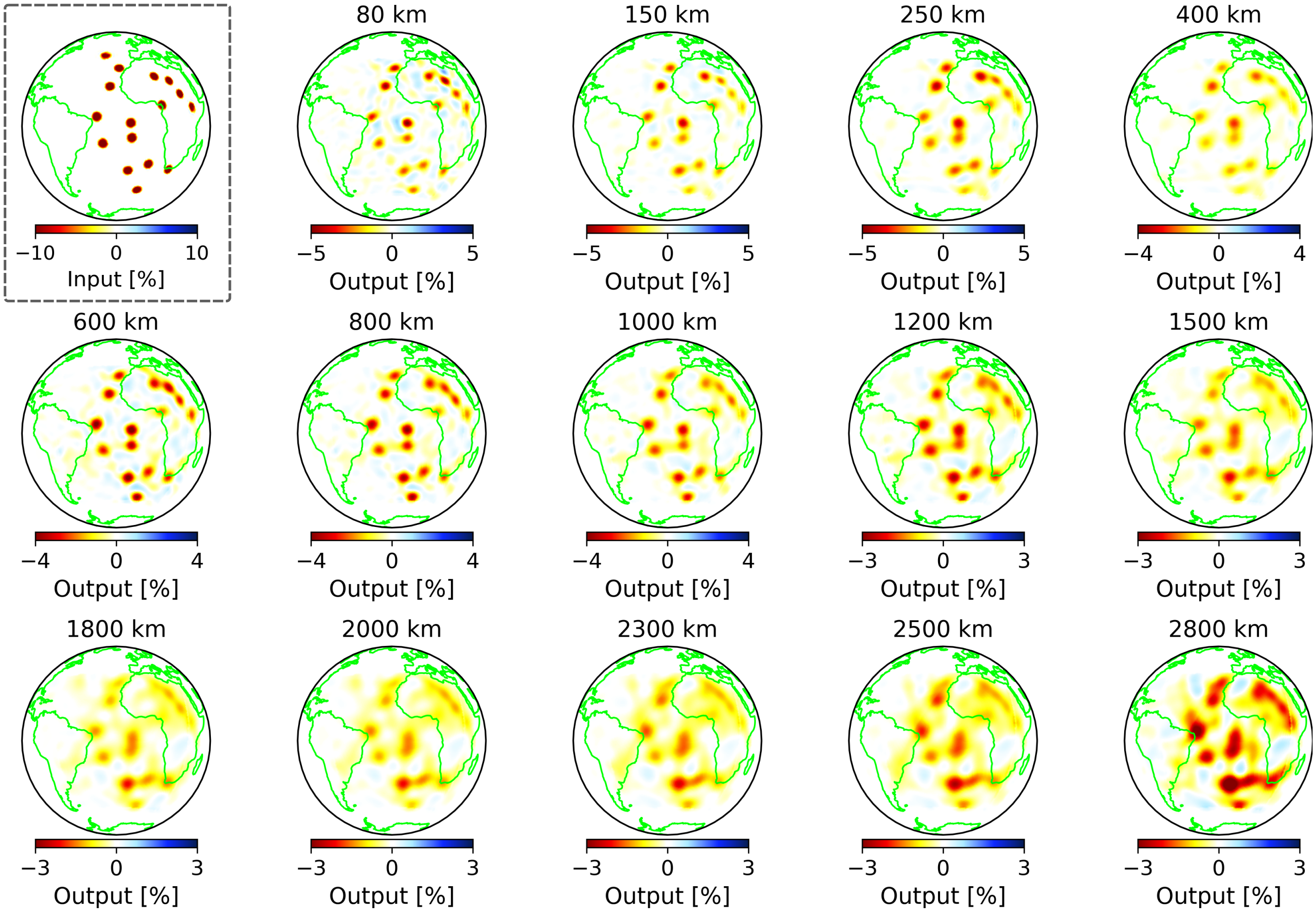

Fig. S7. Linear resolution analysis for the isotropic $\mathrm{V_s}$ portion of the *SEMATL_23* model. Top left panel depicts the input isolated point perturbations (with a radius of 250 km) beneath major hotspots, extending from the core-mantle boundary to the surface. Though amplitudes are not fully recovered, we note good recovery of the morphology of input structures down to roughly 1000 km depth. Below such depths, some of the individual plume-like structures tend to merge, which is why we refer to "plume groups" in the main text. Note however, the three plume groups discussed in the main text remain well-separated from each other all the way down to the CMB.

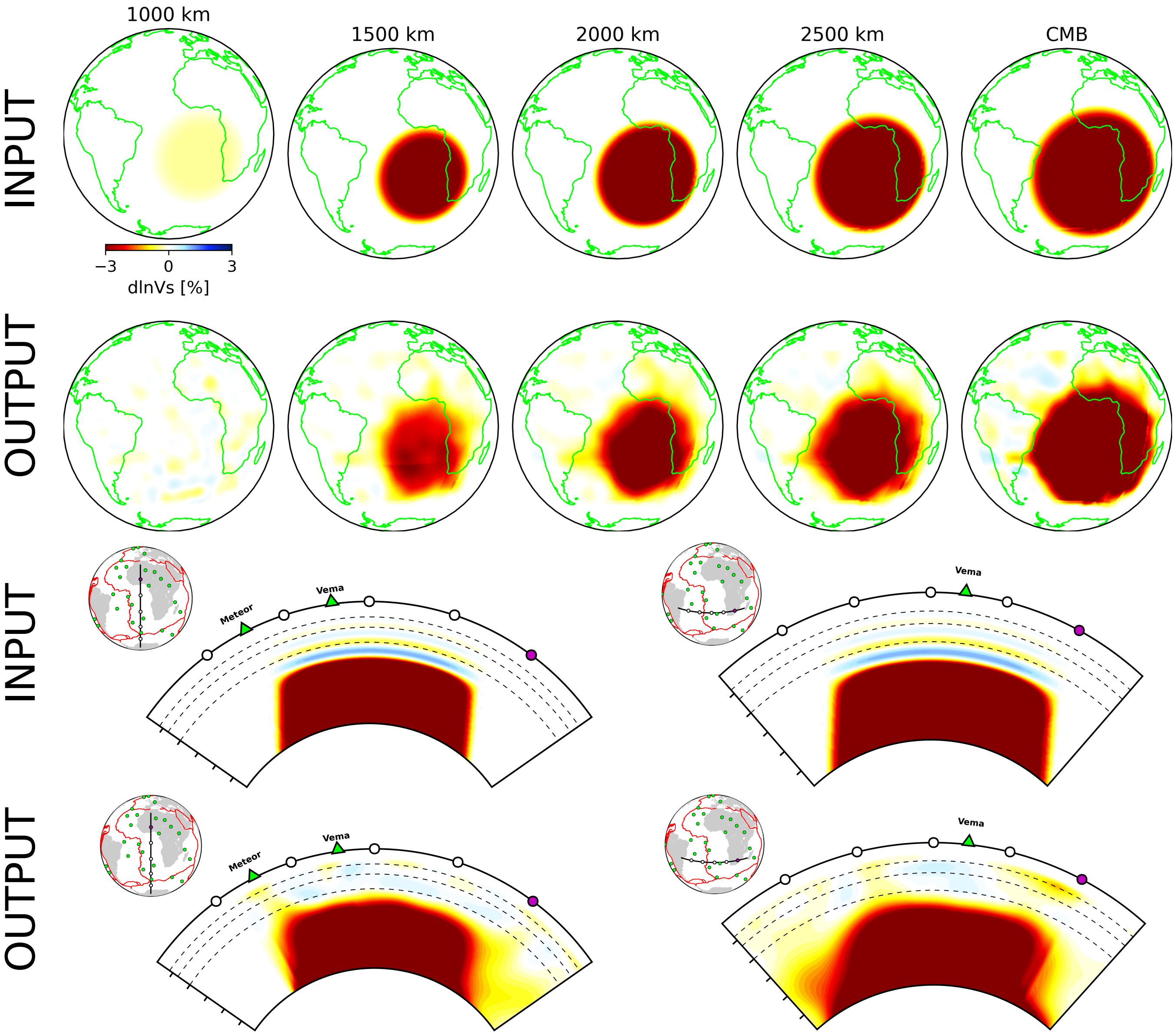

Fig. S8. Linear resolution analysis to test the recovery of a large "LLSVP-like" anomaly in isotropic $\mathrm{V_s}$. Top left panel depicts the input anomaly (with a radius of 2500 km and relative perturbation of 3 %), which extends from the CMB to 1200 km depth (i.e. for a height of ∼1700 km). This test shows that although the amplitude recovered is somewhat reduced at the top of the anomaly, its overall shape is only mildly distorted, and, importantly it is neither broken up into smaller structures, nor smeared to shallower depths.

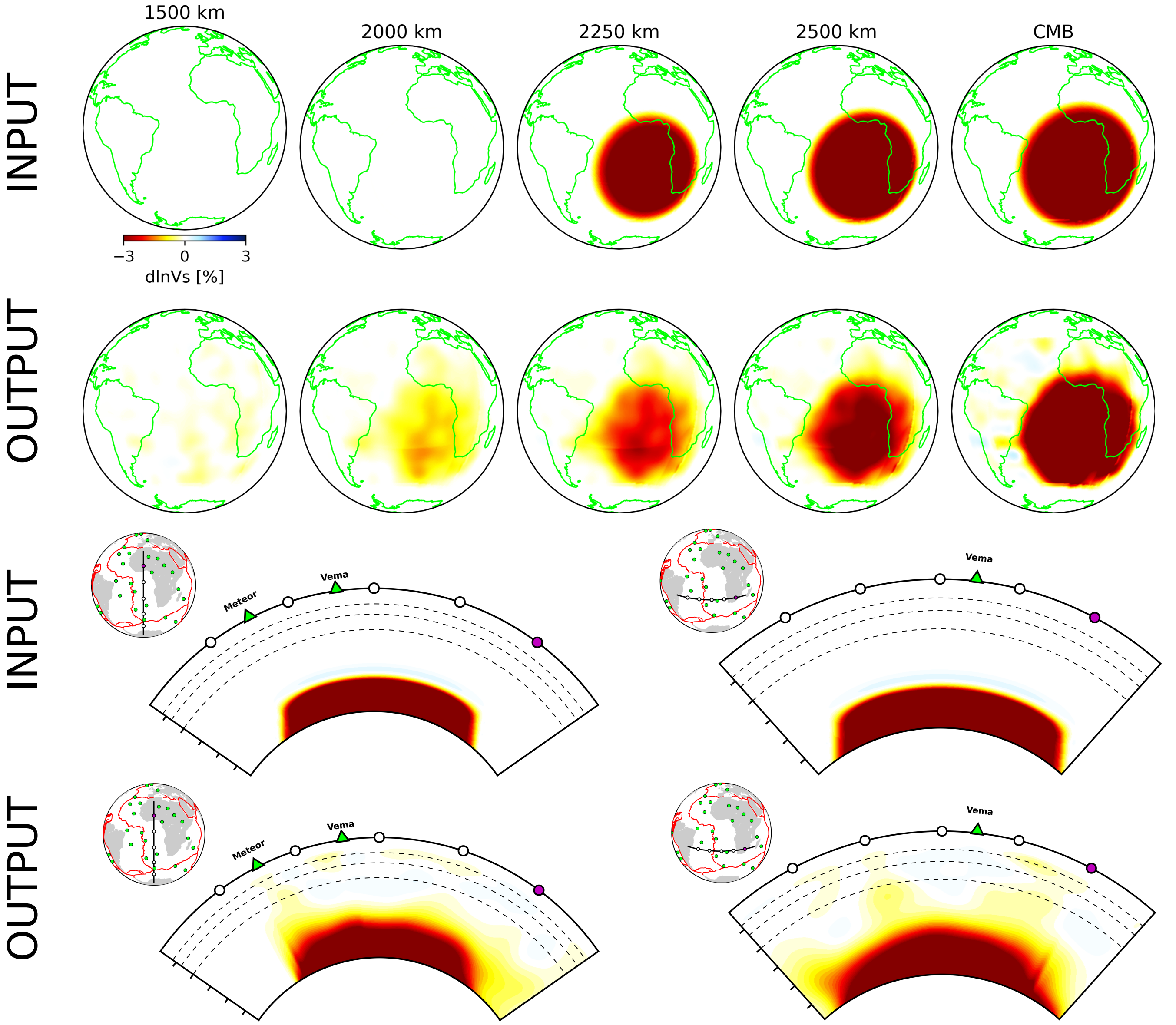

Fig. S9. Same as Fig. S8 for an "LLSVP-like" structure that extends from the CMB to 2000 km depth (i.e for a height of 900 km. Here too the anomaly is not broken up into "plumes" and the smearing at its borders is minimal. The faint plume-like shadows in the mid-lower mantle are an interesting "memory" effect due to the fact that the resolution matrix is assembled in the last iteration leading up to model *SEMATL_23*, a 3D model which contains plume structures. The partial derivatives that enter in the construction of the resolution matrix are computed using NACT, which includes multiple-forward scattering in this 3D model, rather than being referenced to a 1D model. It is not possible to perform a resolution analysis referred to a 1D background model, without redoing the 5 iterations of the model from scratch, a limitation of this resolution analysis, which is still much more efficient than the required analysis of point-spread functions used in adjoint-based full waveform inversions (23).

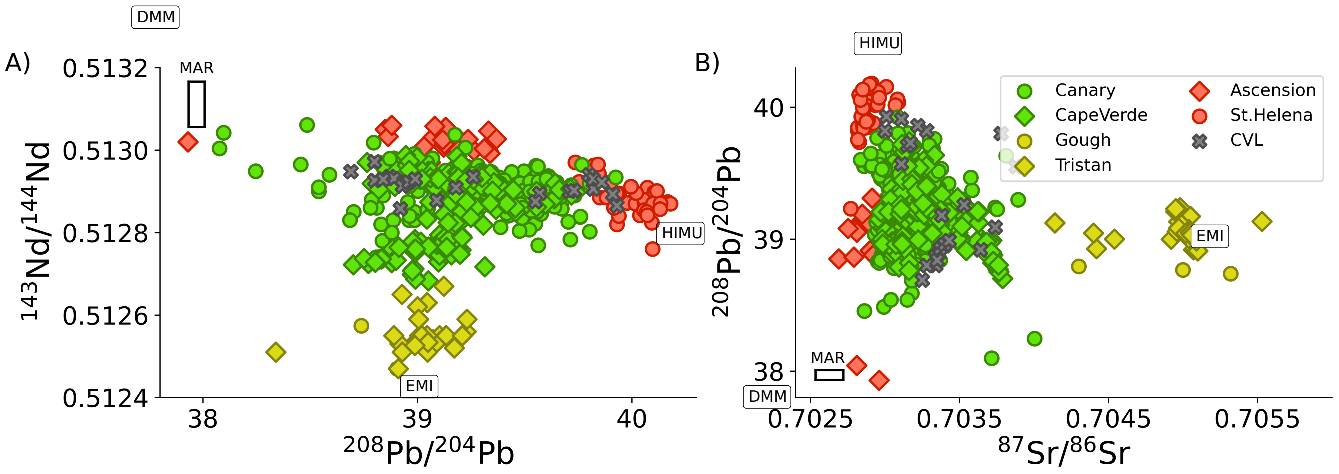

Fig. S10. Radiogenic isotope anomalies in (A) ^{143}Nd/^{144}Nd and ^{208}Pb/^{204}Pb; (B) ^{87}Sr/^{86}Sr and ^{208}Pb/^{204}Pb for major hotspots in the south Atlantic ocean. Colors (green, yellow, and red) indicate similarities in isotopic ratios for different groups of hotspots. Black rectangles represent Atlantic MORB. Gray crosses denote samples from the Cameroon Volcanic Line (CVL).

Table S1. Change in variance reduction (defined as one minus the squared 2-norm of the waveform residual normalized by that of the data) expressed in percentage and number of wavepackets for the initial and final models at each of the two stages in the inversion. The variance reduction increases with iteration as does the number of wavepackets admitted in the inversion, as expected for a successful inversion process.

**Surface waves inversion [Minimum period: 40s]**			
Component	Z	L	T
	Initial model		
Wavepackets	45588	21364	23899
Variance Reduction[%]	67.60	67.92	77.46
	Final model		
Wavepackets	45757	21388	23927
Variance Reduction [%]	70.34	73.14	79.28

**Body waves inversion [Minimum period: 30s]**			
Component	Z	L	T
	Initial model		
Wavepackets	26607	11829	10471
Variance Reduction[%]	71.84	73.67	69.86
	Final model		
Wavepackets	30403	14441	13516
Variance Reduction[%]	79.28	83.44	82.73

References

1. V Lekić, B Romanowicz, Inferring upper-mantle structure by full waveform tomography with the spectral element method. *Geophys. J. Int.* **185**, 799–831 (2011).
2. S French, B Romanowicz, Whole-mantle radially anisotropic shear velocity structure from spectral-element waveform tomography. *Geophys. J. Int.* **199**, 1303–1327 (2014).
3. D Komatitsch, JP Vilotte, The spectral element method: an efficient tool to simulate the seismic response of 2D and 3D geological structures. *Bull. seismological society Am.* **88**, 368–392 (1998).
4. D Komatitsch, J Tromp, Spectral-element simulations of global seismic wave propagation—I. Validation. *Geophys. J. Int.* **149**, 390–412 (2002).
5. D Komatitsch, J Tromp, Spectral-element simulations of global seismic wave propagation—II. Three-dimensional models, oceans, rotation and self-gravitation. *Geophys. J. Int.* **150**, 303–318 (2002).
6. XD Li, B Romanowicz, Comparison of global waveform inversions with and without considering cross-branch modal coupling. *Geophys. J. Int.* **121**, 695–709 (1995).
7. BJ Wood, JR Holloway, A thermodynamic model for subsolidus equilibria in the system CaO–MgO–Al2O3–SiO2. *Geochimica et Cosmochimica Acta* **48**, 159–176 (1984).
8. B Romanowicz, Multiplet-multiplet coupling due to lateral heterogeneity: asymptotic effects on the amplitude and frequency of the Earth's normal modes. *Geophys. J. Int.* **90**, 75–100 (1987).
9. XD Li, B Romanowicz, Global mantle shear velocity model developed using nonlinear asymptotic coupling theory. *J. Geophys. Res. Solid Earth* **101**, 22245–22272 (1996).
10. M Panning, B Romanowicz, A three-dimensional radially anisotropic model of shear velocity in the whole mantle. *Geophys. J. Int.* **167**, 361–379 (2006).
11. JP Montagner, DL Anderson, Petrological constraints on seismic anisotropy. *Phys. earth planetary interiors* **54**, 82–105 (1989).
12. C Mégnin, B Romanowicz, The three-dimensional shear velocity structure of the mantle from the inversion of body, surface and higher-mode waveforms. *Geophys. J. Int.* **143**, 709–728 (2000).
13. Z Wang, F Dahlen, Spherical-spline parameterization of three-dimensional Earth models. *Geophys. Res. Lett.* **22**, 3099–3102 (1995).
14. D Komatitsch, S Tsuboi, J Tromp, The spectral-element method in seismology. *Seism. Earth: Array Analysis Broadband Seism.* pp. 205–227 (2005).
15. Y Capdeville, JJ Marigo, Second order homogenization of the elastic wave equation for non-periodic layered media. *Geophys. J. Int.* **170**, 823–838 (2007).
16. P Cupillard, Y Capdeville, Non-periodic homogenization of 3-D elastic media for the seismic wave equation. *Geophys. J. Int.* **213**, 983–1001 (2018).
17. GE Backus, Long-wave elastic anisotropy produced by horizontal layering. *J. Geophys. Res.* **67**, 4427–4440 (1962).
18. N Shapiro, M Ritzwoller, Monte-Carlo inversion for a global shear-velocity model of the crust and upper mantle. *Geophys. J. Int.* **151**, 88–105 (2002).
19. C Bassin, G Laske, G Masters, The current limits of resolution for surface wave tomography in North America: Eos transactions agu, v. 81, p. *F897* (2000).
20. A Fichtner, BL Kennett, H Igel, HP Bunge, Full seismic waveform tomography for upper-mantle structure in the australasian region using adjoint methods. *Geophys. J. Int.* **179**, 1703–1725 (2009).
21. LW Chen, B Romanowicz, Accelerating full-waveform inversion using source stacking: synthetic experiments at the global scale in a realistic 3-D Earth model. *Geophys. J. Int.* **236**, 644–658 (2024).
22. XD Li, T Tanimoto, Waveforms of long-period body waves in a slightly aspherical Earth model. *Geophys. J. Int.* **112**, 92–102 (1993).
23. B Romanowicz, Global seismic tomography using time domain waveform inversion. *Appl. Data Assim. Inverse Probl. Earth Sci.* **5**, 220 (2023).
24. W Lei, et al., Global adjoint tomography—model GLAD-M25. *Geophys. J. Int.* **223**, 1–21 (2020).
25. NL Celli, S Lebedev, AJ Schaeffer, M Ravenna, C Gaina, The upper mantle beneath the South Atlantic Ocean, South America and Africa from waveform tomography with massive data sets. *Geophys. J. Int.* **221**, 178–204 (2020).
26. B Steinberger, Plumes in a convecting mantle: Models and observations for individual hotspots. *J. Geophys. Res. Solid Earth* **105**, 11127–11152 (2000).
27. M Hoggard, N White, D Al-Attar, Global dynamic topography observations reveal limited influence of large-scale mantle flow. *Nat. Geosci.* **9**, 456–463 (2016).
28. M Holdt, N White, S Stephenson, B Conway-Jones, Densely sampled global dynamic topographic observations and their significance. *J. Geophys. Res. Solid Earth* p. e2022JB024391 (2022).
29. M Seton, et al., A global data set of present-day oceanic crustal age and seafloor spreading parameters. *Geochem. Geophys. Geosystems* **21**, e2020GC009214 (2020).
